# Modeling the influences of climate conditions on measles transmission in China

**DOI:** 10.1017/S095026882510054X

**Published:** 2025-09-11

**Authors:** Peihua Wang, Jianjiu Chen, Wenyi Zhang, Yong Wang, Wan Yang

**Affiliations:** 1 Department of Epidemiology, Mailman School of Public Health, Columbia University, New York, NY, USA; 2 Chinese PLA Center for Disease Control and Prevention, Beijing, China

**Keywords:** measles, climate impact, epidemic dynamics, China, mathematical modeling

## Abstract

Climate conditions are known to modulate infectious disease transmission, yet their impact on measles transmission remains underexplored. In this study, we investigate the extent to which climate conditions modulate measles transmission, utilizing measles incidence data during 2005–2008 from China. Three climate-forced models were employed: a sinusoidal function, an absolute humidity (AH)-forced model, and an AH and temperature (AH/T)-forced model. These models were integrated into an inference framework consisting of a susceptible–exposed–infectious–recovered (SEIR) model and an iterated filter (IF2) to estimate epidemiological characteristics and assess climate influences on measles transmission. During the study period, measles epidemics peaked in spring in northern China and were more diverse in the south. Our analyses showed that the AH/T model better captured measles epidemic dynamics in northern China, suggesting a combined impact of humidity and temperature on measles transmission. Furthermore, we preliminarily examined the impact of other factors and found that population susceptibility and incidence rate were both positively correlated with migrant worker influx, suggesting that higher susceptibility among migrant workers may sustain measles transmission. Taken together, our study supports a role of humidity and temperature in modulating measles transmission and identifies additional factors in shaping measles epidemic dynamics in China.

## Introduction

Measles is a highly transmissible viral disease. Reported basic reproductive number ( 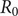 ) for measles ranged from 12 to 18 [1, 2], suggesting on average an infection can cause 12–18 secondary infections in a fully susceptible population. Endemic transmission of measles exhibits varied temporal patterns, ranging from annual [2–4] to bi-annual or multi-annual [5, 6] cycles. These patterns may shift over time and across populations [5–9], likely driven by a combination of factors related to population susceptibility, contact patterns, and climate conditions. For instance, continual replenishment of susceptible individuals driven by high birth rates [5], insufficient vaccination coverage [7], and agricultural labour migration [9, 10] have been shown to help sustain a pool of individuals susceptible to infection and in turn fuel continued transmission. In addition, measles transmission could vary seasonally, due to seasonal variations in population contact patterns, such as school gatherings [5, 6, 11] and, potentially, seasonal changes in climate conditions [2, 12–14].

Climate conditions have been shown to modulate the transmission of respiratory infectious diseases such as influenza [15–18] and respiratory syncytial virus [19–21]. Laboratory experiments have demonstrated that low relative humidity enhances the viability of the measles virus [22, 23], suggesting that climate conditions could similarly affect measles transmission. However, whether and to what extent climate conditions affect measles transmission remains underexplored. It is challenging to examine such influences due to the likely nonlinear climate modulation and the interactions with other contributing factors (e.g., vaccinations and population migration). For instance, previous studies typically relied on statistical analyses (i.e., linear models), which may yield conflicting conclusions [12–14].

In this study, we investigate the potential impact of climate conditions on measles transmission, utilizing a model–inference system and incidence data from China. China is a vast country with diverse climate patterns across its 31 provincial-level administrative divisions (PLADs), which affords the study of measles transmission in the same population but under varying climate conditions. Since 2005, measles vaccination coverage in China has been relatively high (>90% [24]), which helped to curb large epidemics in many municipalities. Nonetheless, epidemics occurred every year in most Chinese PLADs during 2005–2008. From 2009 onward, measles outbreaks in China became more sporadic [25], due to enhanced regional supplementary immunization activities (SIAs) and the 2010 nationwide SIA [26]. Thus, here we used measles incidence data at the PLAD level during 2005–2008, a period with more regular annual epidemics, to focus on examining mechanisms how climate conditions influence measles transmission.

We examined three climate-forced models: 1) a sinusoidal function [4], capturing the annual cycle of measles epidemics; 2) a mechanistic absolute humidity (AH)-forced model [16], assuming transmissibility decreases with specific humidity (a measure of AH); and 3) a mechanistic AH and temperature (AH/T)-forced model [17], assuming a U-shaped relationship with specific humidity and a negative relationship with temperature. Each of these models was integrated into a susceptible–exposed–infectious–recovered (SEIR) model, combined with an iterated filter (IF2) [27], to form an SEIR–IF2 system. We fit each climate-forced model to the PLAD-specific incidence data from 2005 to 2007 to estimate key epidemiological characteristics of measles transmission and generate retrospective forecasts for the year 2008. We assessed the three models and the corresponding climate influences on measles transmission, based on model fit and forecast accuracy.

## Results

### Measles incidence and seasonality

**Figure 1.**
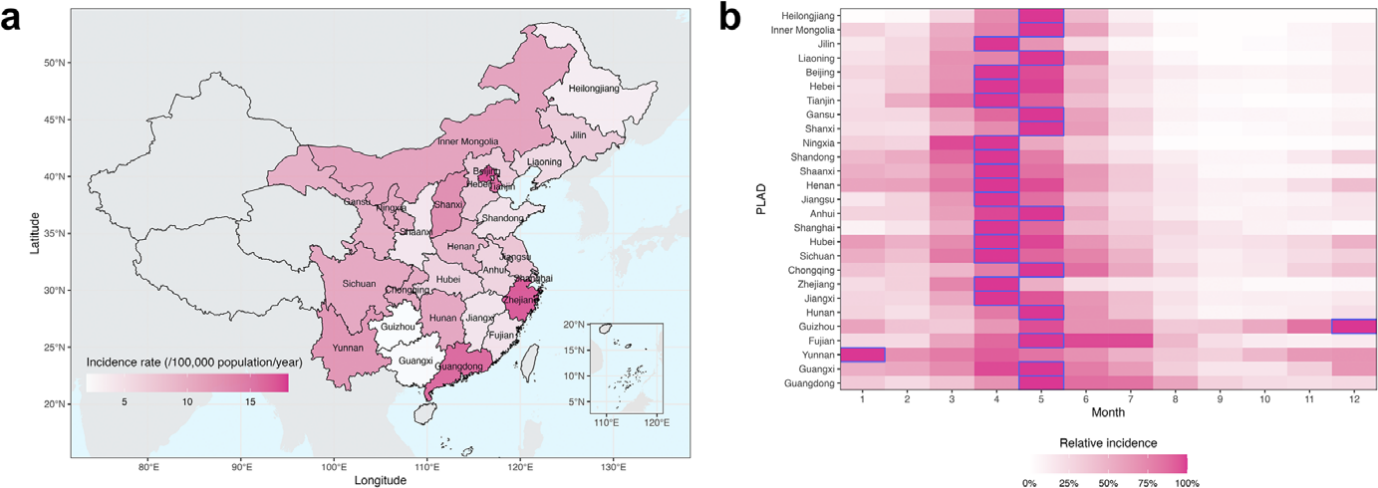
(a) Measles incidence rates and (b) seasonality across PLADs in China, 2005–2008. Heatmap (b) shows the relative incidence, that is, relative to the peak incidence for each PLAD. The boxes indicate the peak months of measles incidence. PLADs on the y-axis are arranged by latitude with higher to lower latitudes from top to bottom.

The average measles incidence rate was 8.83/100,000 population/year from 2005 to 2008 in China. Notably, Beijing, Zhejiang, Guangdong, and Tianjin, some of the most economically developed PLADs, reported the highest incidence rates, exceeding 15/100,000 population/year (Figure 1a). In contrast, Guizhou and Guangxi had the lowest rates, under 3/100,000 population/year. Such higher incidence rates in more developed PLADs could be partly attributed to outbreaks among migrant workers. These populations tended to experience higher infection rates than local residents, likely due to lower vaccination coverage and thus higher susceptibility [28–30]. Additionally, more developed PLADs, equipped with better-resourced healthcare and reporting systems, may have higher reporting rates, which partly resulted in higher reported incidence rates.

During the study period, measles epidemics exhibited pronounced seasonality in northern PLADs, typically surging during winter months and peaking in spring (April and May, Figure 1b). This pattern suggests that seasonal factors such as climate conditions and seasonal fluctuations in human contact patterns may affect measles epidemic dynamics. In comparison, measles epidemics in several southern PLADs displayed less defined seasonality; for example, Guizhou and Yunnan peaked in winter, and Fujian and Guangdong experienced sustained outbreaks throughout spring and summer.

### Validation of SEIR–IF2 system

Prior to applying the SEIR**–**IF2 system to measles incidence data, its ability to estimate underlying epidemiological variables and parameters was tested using synthetic incidence data. The SEIR**–**IF2 system demonstrated reasonable performance with synthetic data. Profile likelihood analyses showed close alignment between parameter values corresponding to high likelihoods and the true parameter values used to generate the synthetic data (note the latent and infectious periods showed minimal sensitivity, as we used narrow prior ranges of 7–9 days and 4–6 days per estimates from the literature [4, 5, 31] for these two parameters, respectively; Supplementary Figures S1a, 2a, and 3a). The IF2 iteration results further showed that IF2 effectively maximized the likelihood across iterations, reproducing observed incidence time series during the inference period, accurately forecasting incidence afterwards, and estimating unobserved state variables (e.g., population susceptibility) and epidemiological parameters (Supplementary Figures S1b, 2b, and 3b).

### Model inference and forecasting of measles epidemic dynamics

**Figure 2.**
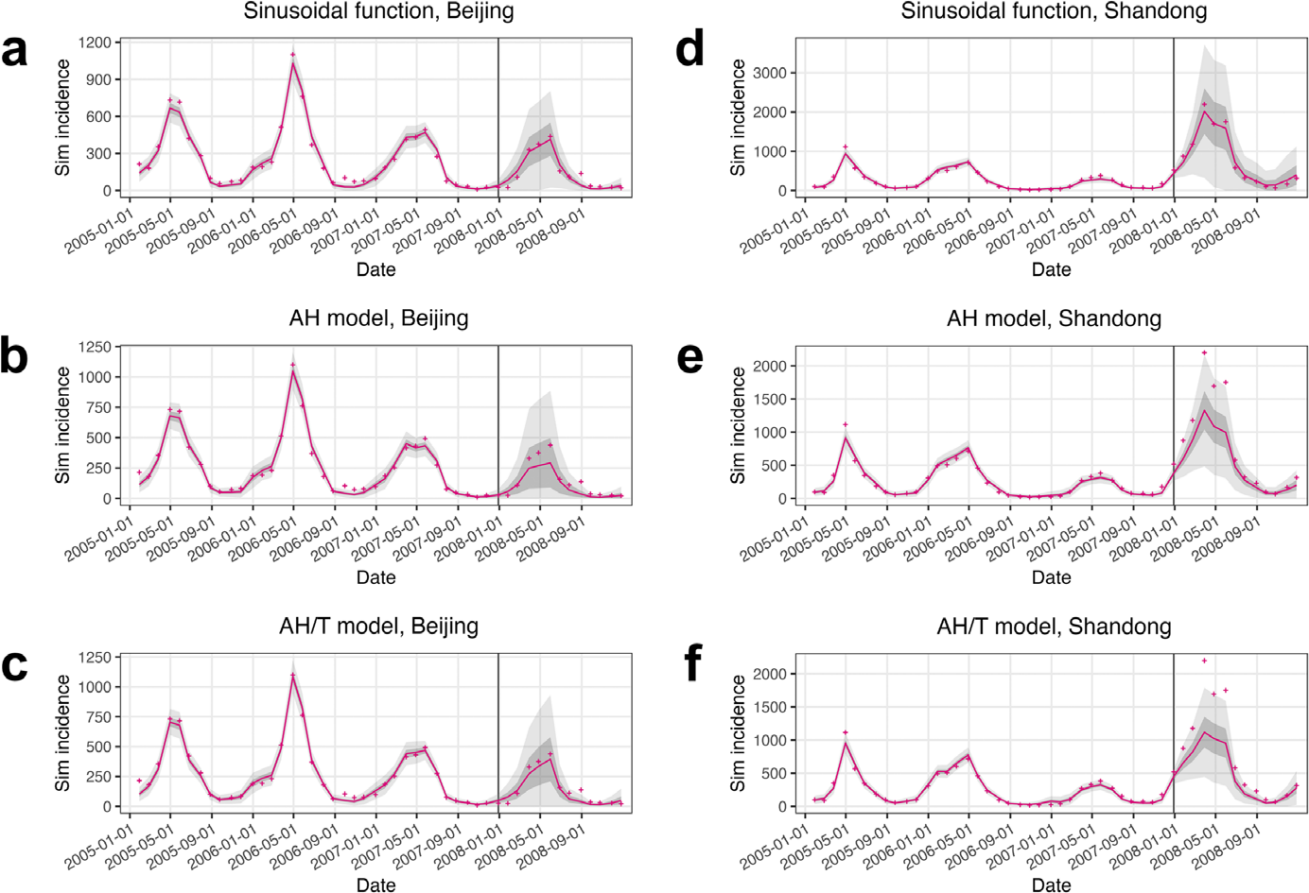
Example model inference and forecasting of measles epidemic dynamics using the sinusoidal function, the AH model, and the AH/T model, for (a, b, c) Beijing and (d, e, f) Shandong. Each plot shows estimated incidence during 2005–2007 and predictions for 2008 (red line indicates mean estimate, dark and light gray areas indicate 50% and 95% credible intervals, and vertical line indicates forecast start), compared to observed incidence (crosses).

PLAD-specific measles incidence data during 2005–2007 were assimilated into the validated SEIR–IF2 system to infer measles epidemic dynamics. The parameter estimates converged by the final iteration (Supplementary Figures S4 and S5), and the estimated incidence closely matched the observations (Figures 2 and Supplementary S6–S11), capturing the annual outbreak pattern that surged during the winter and peaked in the spring. Following the inference period, retrospective forecasts were generated for the year 2008 using the state variable and parameter estimates made at the end of 2007. Predicted incidence generally matched the observations even when the outbreak magnitude during the forecast period differed from that during the inference period (e.g., in Shandong, Figure 2d–f). For instance, the 95% prediction intervals covered 100%, 75%, and 67% of the observed incidence in 2008 using the sinusoidal-, AH-, and AH/T models, respectively.

### Comparison of the climate-forced models

The sinusoidal function demonstrated greater overall effectiveness in capturing measles epidemic dynamics compared to the other two models, based on the ranking of five metrics (see Table 1 for summary statistics, and Supplementary Tables S1 and S2 for individual models and PLADs; see metric definitions in Methods). This is not unexpected, because the phase of the epidemic was treated as a free parameter in the sinusoidal function, rather than determined by climate conditions as in the AH and AH/T models. This flexibility allowed for more accurate prediction of the epidemic peak timing and improved the overall prediction accuracy (see the relative root mean square error (RRMSE) during the forecast period).

**Table 1.** Performance comparison of the climate-forced models

Metric	Sinusoidal function	AH model	AH/T model
AIC_inf prd_	33.8% (1)	32.5% (1)	33.8% (1)
RRMSE_fcast prd_	41.2% (1)	29.4% (1)	29.4% (1)
*r*_fcast prd_	34.4% (1)	34.4% (1)	31.1% (1)
Coverage_fcast prd_	35.8% (1)	34.0% (1)	30.2% (1)
Peak time lag_fcast prd_	40.5% (1)	29.7% (1)	29.7% (1)

Note: Percentage value indicates the percentage of PLADs for which a model yields the highest accuracy under a specified metric (AIC: Akaike Information Criterion; RRMSE: Relative Root Mean Square Error; *r*: correlation coefficient; coverage: proportion of observed incidence falling within the 95% prediction intervals; peak time lag: difference between observed and predicted peak timings). Number in parentheses indicates the ranking of the model performance. The total percentages for each metric may not sum to 100% due to rounding errors.

**Figure 3.**
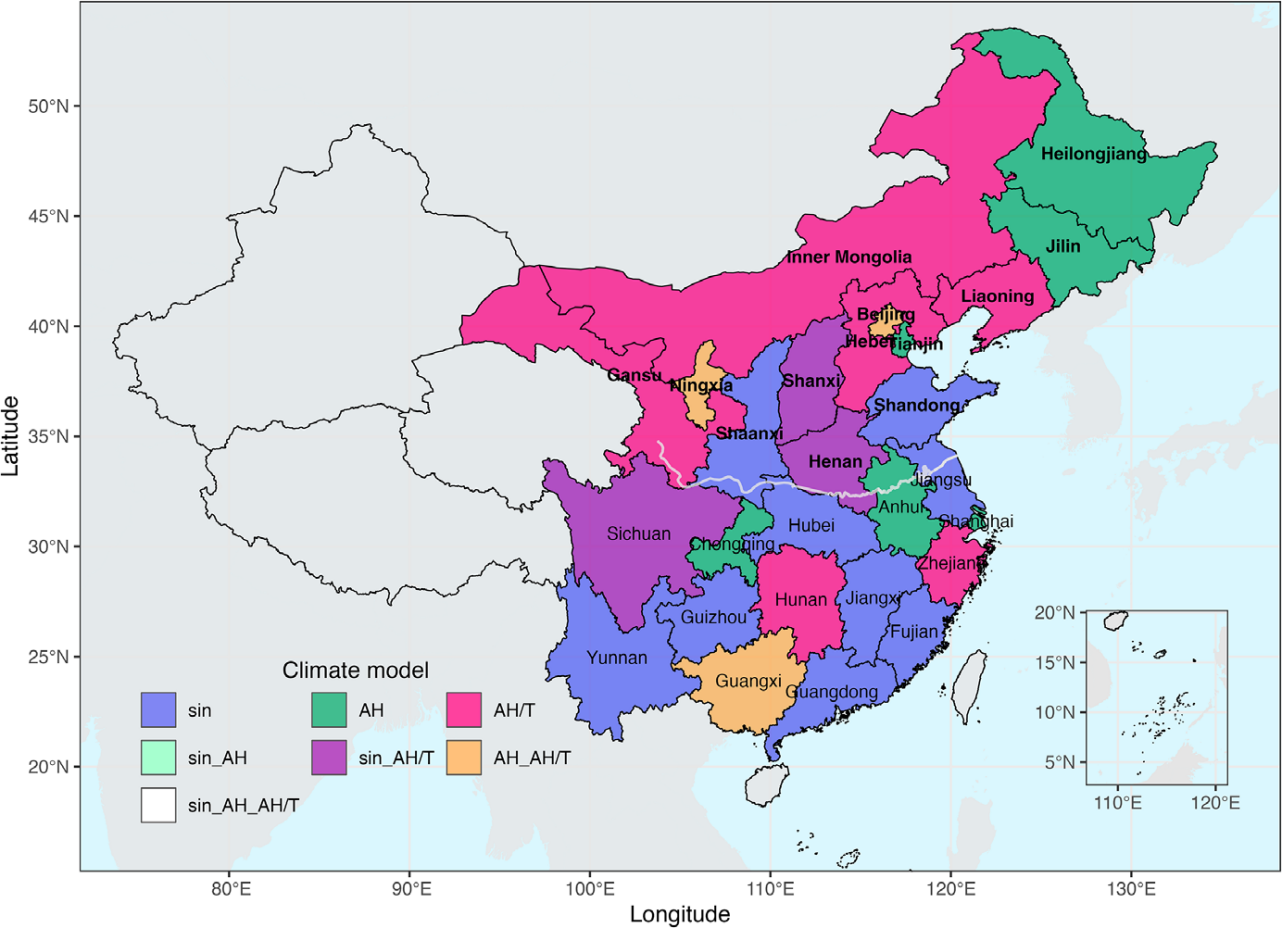
Best-performing models for each PLAD in China. Color indicates the best performing model or models when there are ties (see legend). The gray line indicates the Qin Mountains–Huai River reference line that divides China into northern and southern regions. Bolded fonts indicate northern PLADs.

The AH/T model was able to better capture the measles spring outbreaks in more PLADs in northern China (note we used the conventional northern and southern division based on the Qin Mountains–Huai River reference line), where pronounced epidemic seasonality was observed, compared to the other two models (Figure 3). The sinusoidal function was able to better capture the epidemic dynamics in the south. Southern PLADs experienced more complex epidemic dynamics, as shown by erratic incidence time series in Guizhou (Supplementary Figure S10c), Yunnan (Supplementary Figure S10d), and Guangxi (Supplementary Figure S9e), as well as prolonged outbreaks throughout spring and summer in Fujian (Supplementary Figure S8e) and Guangdong (Supplementary Figure S9d). Additionally, in Guangdong, there was a notable shift in the epidemic peak timing from June to August during the inference period to May during the forecast period. Such variability, not necessarily related to climate conditions, may have reduced the prediction accuracies of the AH and AH/T models in the southern PLADs.

### Estimated impacts of humidity and temperature on measles transmission using the AH/T model

**Figure 4.**
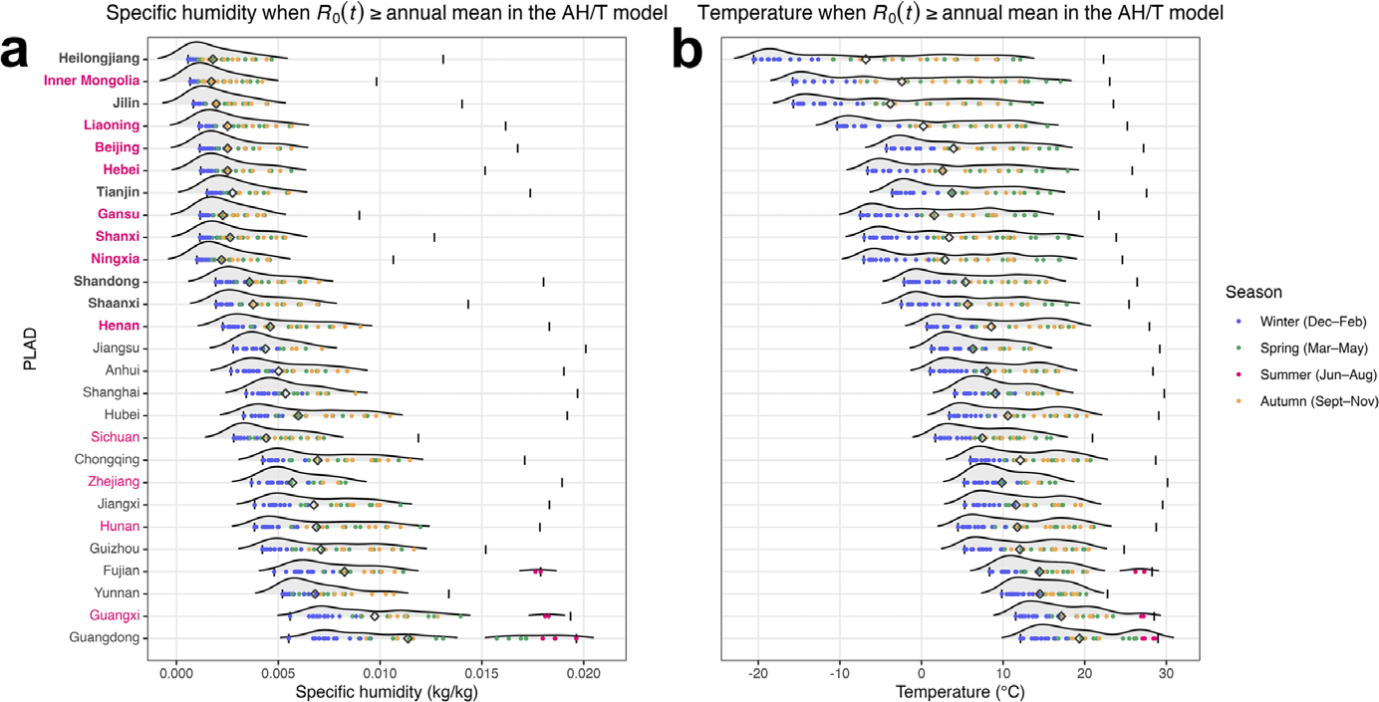
(a) Specific humidity levels and (b) temperatures when 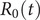 were above the annual mean in the AH/T model, across PLADs in China (diamonds indicate means, and short vertical lines indicate climate condition ranges during the study period, regardless of 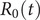 levels). Color of the dots indicates season. PLADs on the y-axis are arranged by latitude with higher latitudes in the top rows. Bolded fonts indicate northern PLADs, and red colors indicate PLADs where the AH/T model was the best-performing model or among the best-performing models.

To examine climate conditions more conducive for measles transmission, we further analyzed specific humidity levels (a measure of AH) and temperatures when the AH/T model estimated 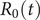 was above or below the annual mean (Figures 4 and S12). In northern PLADs where measles outbreaks peaked in spring, the AH/T model estimated higher-than-average 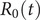 during late autumn, throughout winter, and for most of spring (orange, blue, and green dots, respectively, Figure 4a), a prolonged time period with low specific humidity. In contrast, moderate to high specific humidity during the summer (along with high temperature, Supplementary Figure S12a, b) resulted in lower-than-average 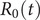 per the AH/T model, which was consistent with the absence of summer outbreaks in the north. In southern PLADs such as Fujian, Guangxi, and Guangdong, in addition to elevated 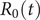 during the winter and spring driven by lower specific humidity as in the north, the AH/T model identified a second period with elevated 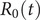 during the summer when specific humidity was very high, that is, a bimodal effect of specific humidity (Figure 4a). This bimodal effect might partly account for the prolonged outbreaks in Fujian and Guangdong (Supplementary Figures S8e and S9d; Guangxi showed irregular outbreaks, Supplementary Figure S9e) during the 2005–2007 inference period. Of note, while the AH/T model did not outperform the sinusoidal function for Fujian and Guangdong, the model fits were comparable (AIC values were 1.5% and 4.4% lower for Fujian and Guangdong, respectively, using the sinusoidal function compared with the AH/T model).

The AH/T model also considered the temperature effect on measles transmission. To assess the importance of modeling this temperature effect, we ran the SEIR**–**IF2 system with a bimodal-AH model, which used the same bimodal humidity component in the AH/T model but without the temperature component. Excluding the temperature component resulted in reduced prediction accuracy, placing it last among the sinusoidal function, the AH model (monotonic dependency), and the bimodal-AH models (Supplementary Table S3). This finding suggests that temperature is also a critical factor in modulating measles transmission, in addition to the bimodal effect of humidity.

Aggregating the climate conditions across the eight northern PLADs for which the AH/T model had the best performance (Figure 4a,b), the AH/T model identified conducive specific humidity levels for measles transmission at 0.0007 to 0.0058 kg/kg, and conducive temperature at −14.1 to 18.6°C (calculated using the 95% confidence intervals of conditions with higher-than-average 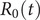 in the eight PLADs). These relatively wide ranges are likely due to the very high infectiousness of measles (i.e., climate conditions may be less limiting for its transmission).

### Examination of the impacts of non-climate-related factors on measles epidemic dynamics

**Figure 5.**
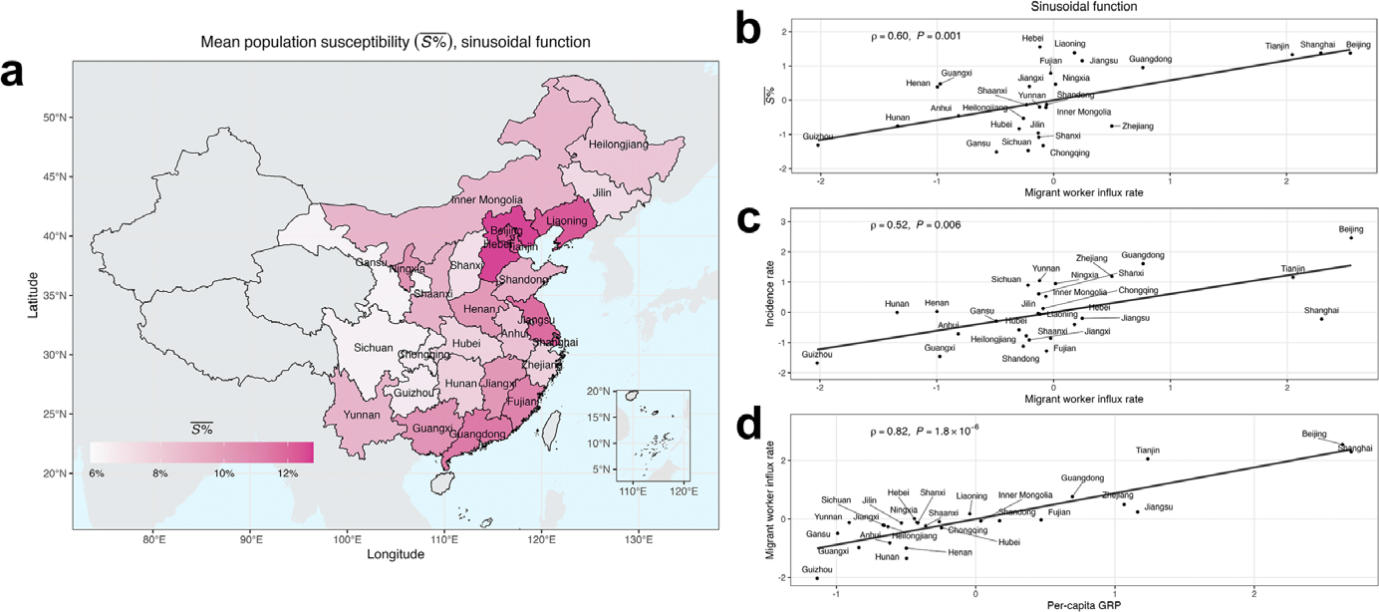
(a) Estimated mean population susceptibility ( 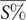 ) during 2005–2007 from the sinusoidal function across PLADs in China. (b) Spearman’s rank correlation between 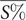 from the sinusoidal function and migrant worker influx rate, 2005–2007. (c) Spearman’s rank correlation between incidence rate and migrant worker influx rate, 2005–2007. (d) Spearman’s rank correlation between migrant worker influx rate and per-capita gross regional product (GRP), 2005–2007. In (b), (c), and (d), standardized variables are presented. Regression lines are included solely for visual guidance.

We further examined the non-climate-related factors based on state variables and parameter estimates. Estimated mean population susceptibility during 2005–2007 (Figures 5a and Supplementary S13; similar estimates using the three climate-forced models) and key epidemiological parameters (Supplementary Figures S14–S16; similar estimates using the three models) aligned with previously reported ranges (Supplementary Table S4). Specifically, the mean population susceptibility was 12.3% (calculated across the three models) in Beijing, 12.2% in Tianjin, 11.8% in Jiangsu, and 7.5% in Zhejiang, in line with serological data [26, 32–34]. Estimated latent period ( 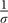 , 8.1 days for Beijing and 7.5 days for Shandong; calculated across the three models for each PLAD), infectious period ( 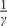 , 5.1 and 4.3 days for the two locations), mixing exponent ( 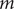 , 0.93 and 0.90), amplitude of school term-time forcing ( 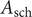 , 0.05 and 0.07), and reporting rate ( 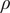 , 58.2% and 72.7%) were comparable to previous modeling results [4].

As noted before, higher population susceptibility of migrant workers likely contributed to the higher reported incidence rates in their host PLADs [28–30]. To test this, we examined the estimated mean population susceptibility and incidence rate with migration statistics across PLADs. Indeed, both quantities were positively correlated with the migrant worker influx rate (calculated as the ratio of the annual influx of migrant workers to the total population; Spearman’s rank correlation test, 
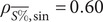
 , 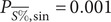 , Figure 5b; similar estimates for the AH and AH/T models; 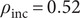 , 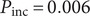 , Figure 5c). That is, despite the higher local immunization rates in host PLADs such as Beijing, Shanghai, and Tianjin (Supplementary Figure S18), estimated population susceptibilities were likely higher due to a large-scale influx of under-vaccinated migrant workers, which increased the overall population susceptibilities in these PLADs. Additionally, the migrant worker influx rate was positively correlated with per-capita Gross Regional Product (GRP; 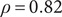 , 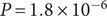 , Figure 5d), indicating that developed PLADs tended to be host PLADs for migrant workers. Taken together, these findings suggest that under-vaccination among migrant workers and their mass migration may increase population susceptibility in more developed PLADs of China, contributing to the higher incidence rates and persistent transmission therein.

Intensified mixing in schools was a key driver of measles transmission in the pre-vaccination era [5]. To reduce transmission risks, China has implemented routine childhood measles vaccination since 1978 [35]. Although detailed data to directly examine the effectiveness of routine vaccination in preventing school outbreaks were unavailable, we examined its impact using a school term-time forcing function included in our models (see Eq. 7 in Methods). We found that PLADs with higher childhood immunization rates had lower estimated amplitudes of school term-time forcing (Spearman’s rank correlation test, 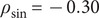 , 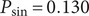 , similar estimates for the AH and AH/T models). This finding suggests that schools were less likely to be the main sources of measles infection in PLADs with higher childhood immunization rates.

## Discussion

In this study, we investigated the climate influences on measles transmission through mechanistic modeling. Analyzing incidence data spanning 27 PLADs in China using three climate-forced models, we found that the mechanistic AH/T model was able to better capture the spring outbreaks in northern PLADs, where pronounced epidemic seasonality was observed. Additionally, the bimodal effect of humidity might partly account for the seasonality of summer outbreaks observed in certain southern PLADs. These findings suggest the bimodal effect of humidity, in conjunction with temperature, in modulating measles transmission. Analysis of inference results and demographic patterns also showed positive correlations of population susceptibility and incidence rate with migrant worker influx, as well as an inverse relationship between childhood immunization rate and the amplitude of school term-time forcing. These findings suggest substantial influences of migrant workers due to their high population susceptibility on sustaining measles transmission and the effectiveness of routine childhood measles vaccination in reducing the risk of school outbreaks.

The AH/T model provides a more accurate representation of the mechanisms that both the bimodal effect of specific humidity and temperature modulate measles transmission. The AH/T model was originally developed to capture the biannual epidemics or less defined seasonality of influenza in subtropical and tropical regions [17, 36]. In our study, it also accurately predicted the spring outbreaks of measles in temperate northern PLADs in China, where it estimated elevated 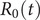 from late autumn to spring driven by low humidity. In subtropical southern PLADs such as Fujian and Guangdong, the model identified a second period of elevated 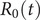 during the summer, when very high humidity occurred (Figure 4a), which might in part explain the summer outbreaks in these southern PLADs (Supplementary Figures S8e and S9d). Additionally, the reduced prediction accuracy observed after excluding the temperature component from the AH/T model underscores the role of temperature in modulating measles transmission (Supplementary Table S3).

Unexpectedly, the AH/T model did not perform as well in the subtropical southern PLADs. More irregular measles outbreaks were observed in southern PLADs such as Guizhou (Supplementary Figure S10c), Yunnan (Supplementary Figure S10d), and Guangxi (Supplementary Figure S9e), potentially due to non-climate-related factors; for instance, mountainous terrain and more limited economic development may impede access to medical clinics [37, 38] and in turn case reporting, which could affect accuracy of the surveillance systems and obscure the underlying epidemic dynamics. In Guangdong, another southern PLAD, the epidemic peak timing shifted from summer during the 2005–2007 inference period to spring during the 2008 forecast period. The reasons for this delayed epidemic peak timing and the shift are not clear and likely include factors other than climate conditions (see discussion below).

Beyond climate influences, worker migration may affect the measles epidemic dynamics in China by altering population susceptibility in migrant worker host PLADs. During the study period, migrant workers accounted for a substantial proportion of China’s population (5.7% [39]), and much higher proportions in host PLADs (e.g., 29.5% in Beijing [39]). These populations tended to be under-vaccinated due to limited healthcare access in their home regions and typically moved to economically developed PLADs shortly after the Chinese New Year holiday in February to seek employment. This large-scale seasonal migration could rapidly alter population susceptibility in host PLADs, contributing to the spring outbreaks therein. This finding is consistent with epidemiological studies in Beijing [28, 29] and Shanghai [30]—two of the most developed PLADs in China – which reported a notable rise in measles incidence primarily among migrant worker populations with lower vaccination coverage during our study period. Our previous modeling work [3, 31] also pointed to the role of worker migration, particularly around the Chinese New Year period when travel was most intense, in sustaining measles transmission. Furthermore, potential seasonal migration of ‘left-behind children’ – that is, children of migrant workers who were left in their hometowns [40, 41] – could complicate measles epidemic dynamics. During school summer breaks, some of these children traveled to urban centers for family reunions [42]. This summer migration may in part explain the measles outbreaks in Guangdong (a PLAD ranked #1 in the number of migrant workers [39]) between June and August. Future work could further incorporate these factors to improve the accuracy in estimating population susceptibility and key epidemiological parameters.

Our study has several limitations. One is that, for simplicity, our SEIR model did not account for age-structured social mixing patterns. Additionally, our results on the impact of migrant workers, based on the correlation of estimated population susceptibility and migrant worker influx rate, were preliminary. Work to explicitly model worker migration incorporating migration and population mobility data is underway in our team and should provide stronger, causal support to the impact of worker migration on measles epidemic dynamics in China. Our study also has several strengths. By examining three climate-forced models against incidence data across 27 Chinese PLADs with diverse climate conditions and measles epidemic dynamics, our results indicate that: 1) both humidity and temperature modulate measles transmission; 2) higher population susceptibility of migrant workers contributes to sustained measles transmission; and 3) routine childhood measles vaccination reduces the risk of school outbreaks. These findings suggest that improving vaccination coverage among migrant workers and maintaining high childhood vaccination coverage including among migrant children are crucial to curb measles transmission, particularly in large cities with substantial migrant worker populations. By testing several mechanistic models that incorporate different effects of humidity and temperature, either alone or in combination, we were able to examine their roles separately. Our findings support a role of both humidity and temperature in modulating measles transmission.

## Methods and materials

### Study data

Incidence data in China were sourced from the Data Centre of China Public Health Science [43]. Demographic data, including birth and death rates, the sizes of total and migrant worker populations, the numbers of primary-school students and primary schools, and per-capita GRP were obtained from the National Bureau of Statistics of China [44], the China Population and Employment Statistics Yearbook [39], the China City Statistical Yearbook [45], and the Tabulation on the 2010 Population Census of the People’s Republic of China [46]. Climate data, including specific humidity and temperature, were obtained from the Integrated Surface Dataset maintained by the National Oceanic and Atmospheric Administration [47]. The data show minimal climate variability among cities within the same PLAD (Supplementary Figure S17; Sichuan shows slightly higher variability).

Immunization rate for two doses of measles vaccine was calculated as 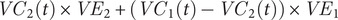 , where 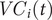 is the vaccination coverage for the 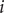 -th dose in year 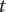 , and 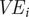 is the vaccine effectiveness for the 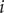 -th dose. 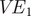 and 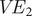 were assumed to be 85% and 95%, respectively [31]. Of the 31 PLADs, two (Beijing [4] and Guizhou [48]) reported vaccination coverages for both doses, and eight [49–56] reported coverage for only one dose; further, data were available only for certain years between 2005 and 2014. To estimate missing vaccination coverage for the other dose (i.e., 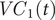 or 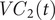 ) for the eight PLADs, we first estimated the series completion rates ( 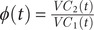 ), based on logistic functions fitted to data from Beijing and Guizhou (with both 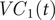 and 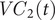 for certain years) between 2005 and 2014 ( 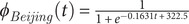 , 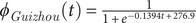 , midpoints ≤1990). Given that the series completion rate is likely related to economic development and healthcare access, we estimated these rates for the eight PLADs using a linear regression model based on their per-capita GRP rankings for each year (note that Beijing and Guizhou ranked 2nd and 31st, respectively, during 2005–2014), and computed the missing vaccination coverages accordingly. Subsequently, for years with reported and estimated vaccination coverage data, immunization rates combining both doses were computed for Beijing, Guizhou, and the eight PLADs. Missing immunization rates between 2005 and 2014 were then estimated using the logistic functions with the same mathematical form as above. For the PLADs without publicly available vaccination coverage data, immunization rates were imputed using a linear regression model based on per-capita GRP rankings for each year. Immunization rate estimates from 2005 to 2008 were used as model inputs (Supplementary Figure S18).

As noted in the Introduction, due to high vaccination coverage [24], measles outbreaks were more sporadic at the city level from 2005 to 2008. As such, PLAD-level measles incidence data from 2005 to 2008 were used. The data were divided into two parts: an inference period from 2005 to 2007 and a forecast period in 2008. Among the 31 PLADs in mainland China, 27 were included in this study. Hainan, Tibet, Qinghai, and Xinjiang were excluded due to their incidence time series showing no clear temporal patterns (Supplementary Figure S19).

### Basic SEIR model

The transmission dynamics of measles were simulated using a stochastic SEIR model with a daily time step. The basic form of the model is governed by the following differential equations:
(1)

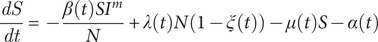



(2)

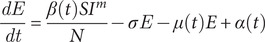



(3)

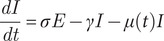



Here, 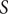 , 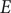 , 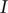 , and 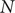 are the susceptible, exposed, infectious, and total population sizes, respectively, with 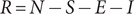 representing those recovered and/or immunized. 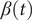 is the time-varying transmission rate, 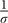 and 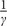 are the latent and infectious periods, respectively, and 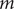 indicates the degree of inhomogeneous mixing [5]. 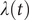 and 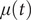 are the birth and death rates, respectively. 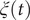 is the immunization rate of routine vaccination in infants. 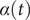 represents travel-related case importations, which was modeled as a Poisson distribution (the mean rate was set to 0.01/day to allow case reintroduction and prevent epidemic extinction, and it increased to 0.1/day during the Chinese New Year and National Day holidays to account for increased travel activities [4]). Transition rates (between model state variables, namely, 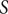 , 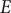 , *I*, and *R*) were drawn from Poisson distributions with mean rates determined by Eqs. 1–3 to simulate stochastic transmission dynamics.

### Climate-forced model

Three climate-forced models, namely the sinusoidal function [4], the AH model [16], and the AH/T model [17], were used to model seasonal changes in the daily basic reproductive number ( 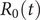 ) of measles. The sinusoidal function models 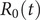 independent of climate data [4]:
(4)

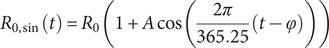



Here, 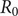 is the annual mean of 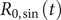 , and 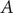 and 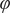 are the amplitude and phase of the sinusoidal function, respectively.

The AH model, initially developed for modeling influenza epidemics in temperate regions, models 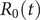 as an exponentially decreasing function of specific humidity [16]:
(5)

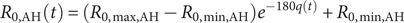



Here, 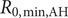 and 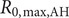 are the minimum and maximum values of 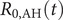 , respectively, and 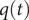 is the daily specific humidity.

The AH/T model, initially developed for modeling influenza epidemics in tropical and subtropical regions, models 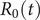 as a convex quasi-quadratic function of specific humidity multiplying an inverse power function of temperature [17]:
(6)

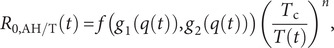


where 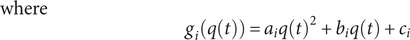



Here 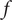 is the quasi-quadratic function, and 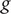 is the quadratic function. 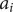 , 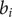 , and 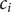 are calculated from three specific points: 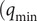 , 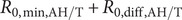 ), ( 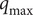 , 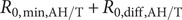 ), and ( 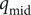 , 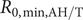 ). 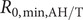 and 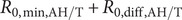 are the minimum and maximum values of the quasi-quadratic function, respectively. 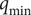 and 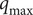 are the upper and lower limits of the specific humidity permitted in the model, respectively, and 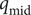 is the specific humidity within the range of ( 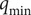 , 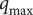 ) where 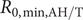 is estimated. 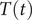 is the daily temperature, and 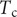 is the cutoff temperature. 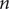 modifies the degree of the impact of temperature on 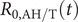 .

The three climate-related functions are further modified to account for the increased contact rates among children during school terms [57]:
(7)

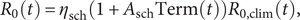


where 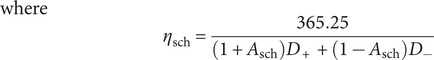



Here, 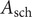 is the amplitude of school term-time forcing. 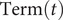 is set to +1 during school terms and − 1 during winter and summer breaks. 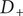 and 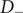 are the number of days in school terms and breaks, respectively. 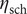 is a normalization factor that ensures the school term-time forcing, 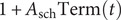 , averages to 1 over the course of 1 year. 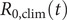 is the climate-related component of 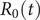 , that is, 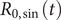 , 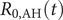 or 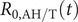 depending on the climate forcing.


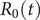
 relates to 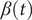 in Eqs. 1–2 of the basic SEIR model through the following expression:
(8)

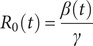



### SEIR–IF2 system

The climate-forced SEIR model, in conjunction with IF2 [27], forms the SEIR**-**IF2 system to estimate unobserved model state variables and parameters during 2005–2007. IF2, a data assimilation technique, uses an iterated, perturbed Bayes map to iteratively refine these estimates based on real-world observations. Within this system, each iteration of IF2 consists of parameter perturbations and a particle filter (PF) [58] to assimilate measles incidence time series. The resulting parameters from one iteration serve as initial parameters for the subsequent iteration. This iterative process is designed to converge on state variables and parameters that maximize the likelihood of reproducing the entire incidence time series. In this study, the SEIR**-**IF2 system underwent 50 IF2 iterations to maximize information extraction from the 3-year dataset. Compared to a single-pass PF, equivalent to IF2 with one iteration (Supplementary Figure S1b, iteration number = 1), IF2 showed improved accuracy in estimating stable variables and parameters.

In each IF2 iteration, the PF estimated the unobserved state variables and parameters ( 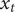 ) of the climate-forced SEIR model given the observations ( 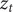 ). 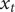 encompasses model state variables ( 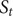 , 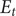 , 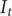 ), common parameters ( 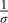 , 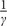 , 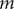 , 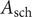 , 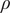 ), and specific parameters for the three climate-forced models: ( 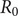 , 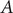 , 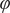 ) for the sinusoidal function, ( 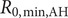 , 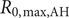 ) for the AH model, and ( 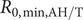 , 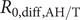 , 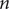 ) for the AH/T model. 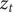 represents observed incidence. Other parameters were treated as constants (see parameter values in Supplementary Table S5). Specifically, the PF calculates the posterior distribution of 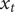 given observations 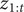 , expressed as 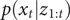 , in a recursive manner [58]:
(9)

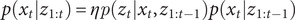




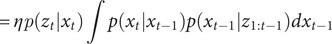



Here, 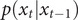 is the model prior computed by the climate-forced SEIR model. 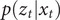 is the observation model; here, it was modeled using a normal distribution 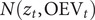 with the observation error variance ( 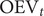 ) adjusted according to the magnitude of 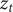 . 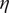 is a normalization constant.

To account for model stochasticity, 20 independent runs were performed. The estimates from these runs were then combined according to Rubin’s rule [59].

### Initialization of the SEIR–IF2 system

The SEIR**–**IF2 system was initialized with 10,000 particles, collectively representing particle approximated initial state variables and parameters ( 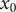 ). Among the model state variables and parameters, prior ranges of susceptible population ( 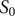 ), reporting rate ( 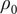 ), and mixing exponent ( 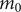 ) were estimated from incidence, birth, and total population data using a time series susceptible**–**infected**–**recovered (TSIR) model [5] and the R package tsiR (v0.4.3) [60]. This model offers a more informed approach for setting initial conditions compared to random sampling from a broad range. Prior ranges for other model state variables and parameters were informed by estimates in the literature [1, 4, 17, 61], and are listed in Supplementary Table S6.

### Incidence forecasting

We generated predictions of measles incidence in year 2008, using state variable and parameter estimates made at the end of 2007 from the SEIR**–**IF2 system.

### Validation of SEIR–IF2 system

The SEIR**–**IF2 system was validated using synthetic incidence data. The synthetic incidence data were generated using each of the climate-forced stochastic SEIR models and climate data in Beijing (see prescribed parameter values in Supplementary Figures S1 **–S** 3; mean of 10,000 simulations used). The procedures of model inference and forecasting described in the above sub-sections were then applied to the synthetic data. The estimated state variables and parameters were compared to the prescribed true values to assess the effectiveness of the SEIR**–**IF2 system.

### Performance comparison of climate-forced models

The performance of the climate-forced models was assessed using five metrics. For the inference period, the Akaike Information Criterion (AIC) [62] was used. For the forecast period, four metrics were used: 1) relative root mean square error (RRMSE) between the predicted and observed incidence; 2) correlation coefficient (*r*) between the predicted and observed incidence; 3) coverage, calculated as the proportion of observed incidence falling within the 95% prediction intervals; and 4) peak time lag, calculated as the observed peak timing minus the predicted peak timing. We determined the best performing model or models (in case of ties) for each PLAD, based on the overall ranking across the five metrics. The ranking method awarded votes to models with the highest accuracy per each metric (e.g., lowest RRMSE or highest *r*) and those within a tolerance threshold of the highest accuracy. The tolerance thresholds for the five metrics were set at 5%, 0.05, 0.05, 0.5, and 5%, respectively. The model or models receiving the highest number of votes were deemed the best. Sensitivity analyses that adjusted the thresholds to 0.6 and 1.4 times of the values listed above gave consistent rankings and confirmed the robustness of the ranking method.

## Supporting information

10.1017/S095026882510054X.sm001 Wang et al. supplementary material Wang et al. supplementary material

## Data Availability

The measles incidence data are subject to restriction. To access these data and/or to seek permission for its use, please contact the Data-center of China Public Health Science (https://www.phsciencedata.cn/Share/).

## Supplementary material

The supplementary material for this article can be found at http://doi.org/10.1017/S095026882510054X.
