# Modeling the Influences of Climate Conditions on Measles Transmission in China – ERRATUM

**DOI:** 10.1017/S0950268825100745

**Published:** 2025-11-07

**Authors:** Peihua Wang, Jianjiu Chen, Wenyi Zhang, Yong Wang, Wan Yang

**Affiliations:** 1 Department of Epidemiology, Mailman School of Public Health, Columbia University, New York, NY, USA; 2 Chinese PLA Center for Disease Control and Prevention, Beijing, China

When this article was originally published in Epidemiology & Infection it contained errors in Table 1, which were introduced during the article’s production. This has been updated in the original article.

The publisher apologises for this error.
