# Supplementary material for: Modeling the influences of climate conditions on measles transmission in China
Source: Epidemiol Infect. 2025 Sep 11;153:e110. doi: 10.1017/S095026882510054X (PMC12529438; doi:10.1017/S095026882510054X)
Supplement: Wang et al. supplementary material [file S095026882510054Xsup001.docx]

**Supplementary Information**

**Modeling the Influences of Climate Conditions on Measles Transmission in China**

Peihua Wang^1,*^, Jianjiu Chen^1^, Wenyi Zhang^2^, Yong Wang^2^, Wan Yang^1,*^

^1^ Department of Epidemiology, Mailman School of Public Health, Columbia University, New York, New York, United States

^2^ Chinese PLA Center for Disease Control and Prevention, Beijing, China

(*) Corresponding authors:

Peihua Wang (pw2586@cumc.columbia.edu)

Wan Yang (wy2202@cumc.columbia.edu)


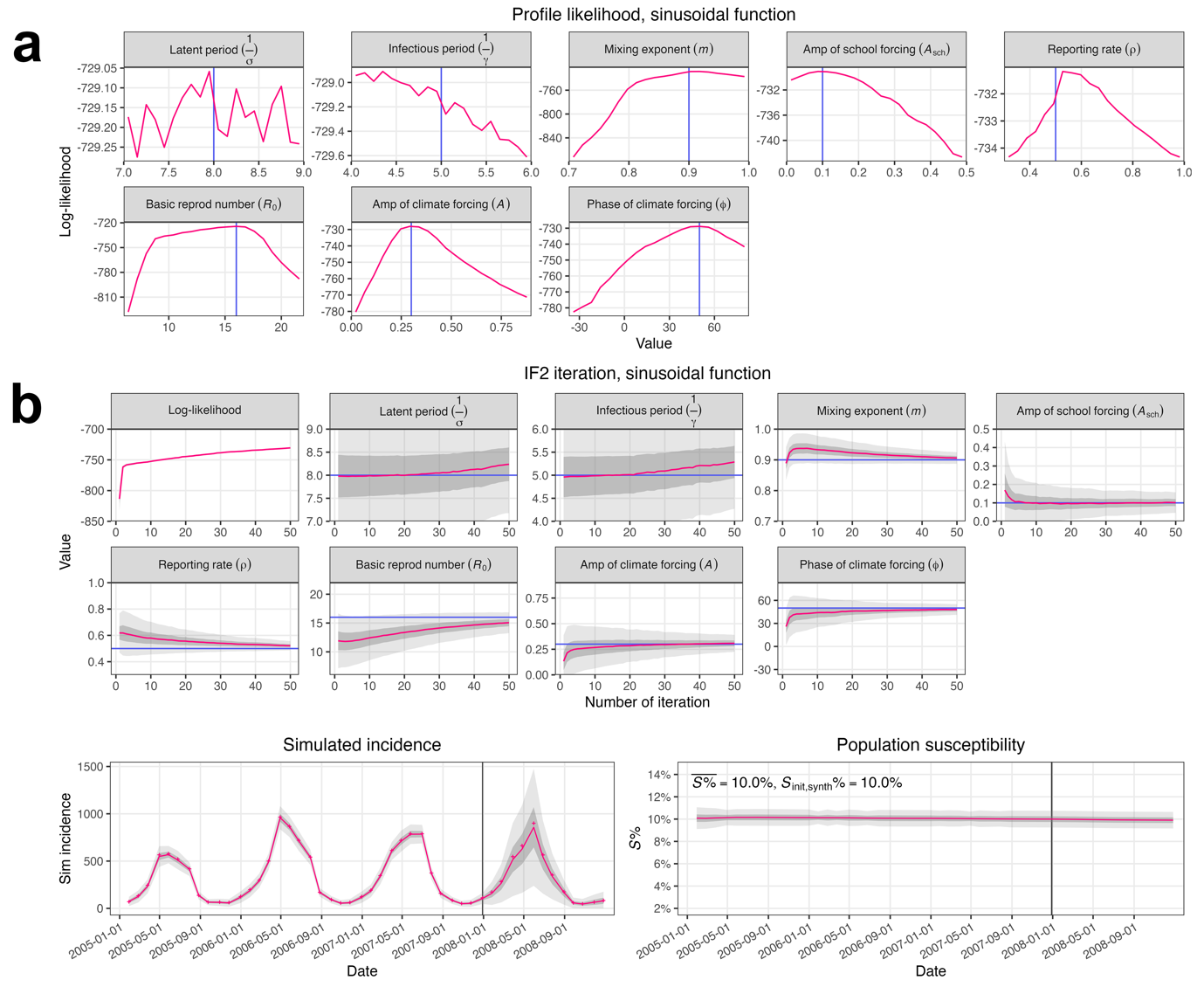


**Fig. S1** Validation of the SEIR–IF2 system using the sinusoidal function. (**a**) Profile likelihoods for model parameters (blue lines indicate prescribed parameter values, and red lines indicate mean estimates). (**b**) IF2 iteration results. Upper subplots show IF2 convergence on parameters that maximize likelihood (dark and light grey areas indicate 50% and 95% credible intervals). Lower left subplot shows estimated incidence during 2005–2007 and predictions for 2008 (vertical line indicates forecast start), compared to the synthetic data (crosses). Lower right subplot shows estimated population susceptibility ($S\%$, prescribed initial $S\%=10.0\%$).


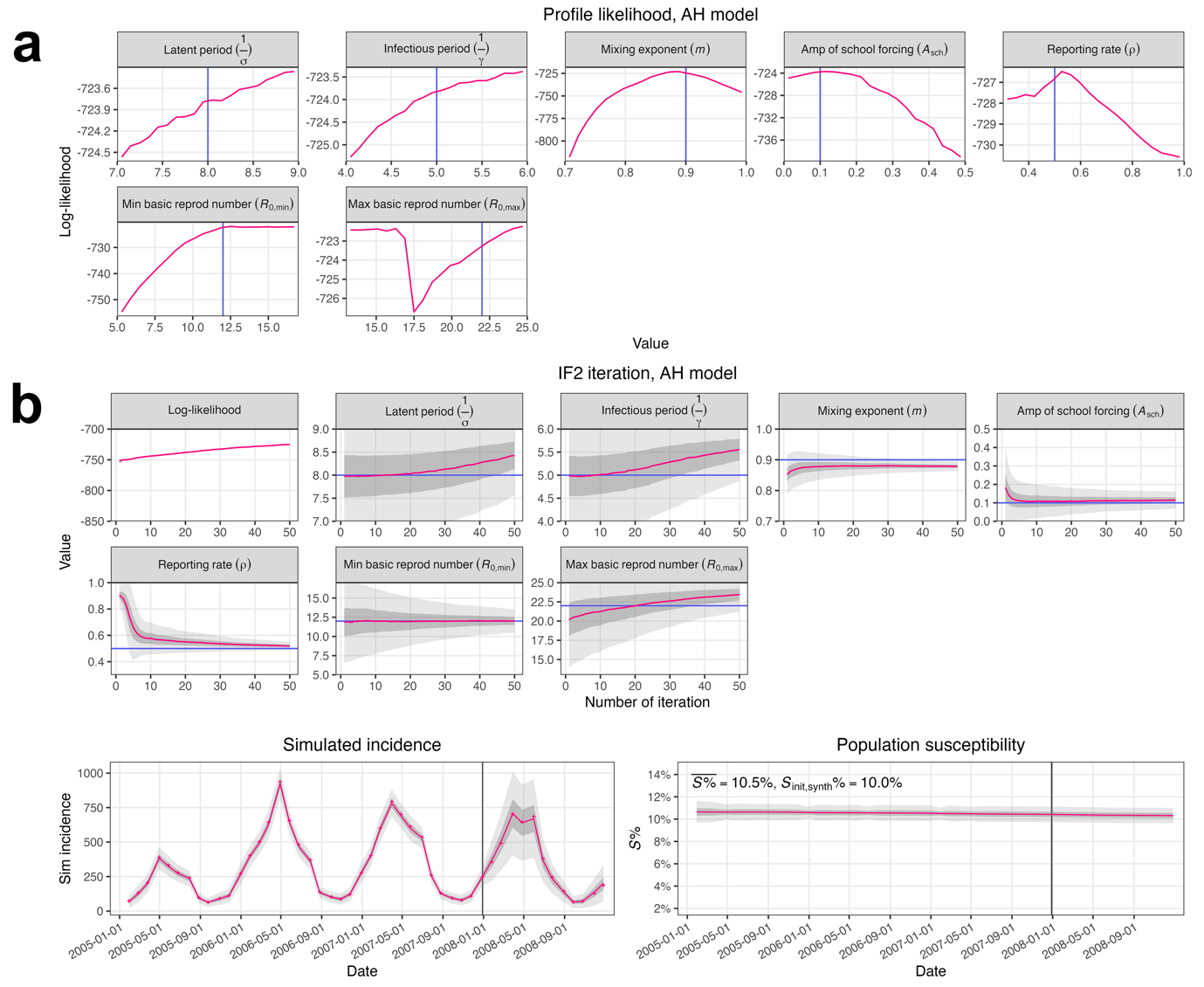


**Fig. S2** Validation of the SEIR–IF2 system using the AH model. (**a**) Profile likelihoods for model parameters. (**b**) IF2 iteration results.


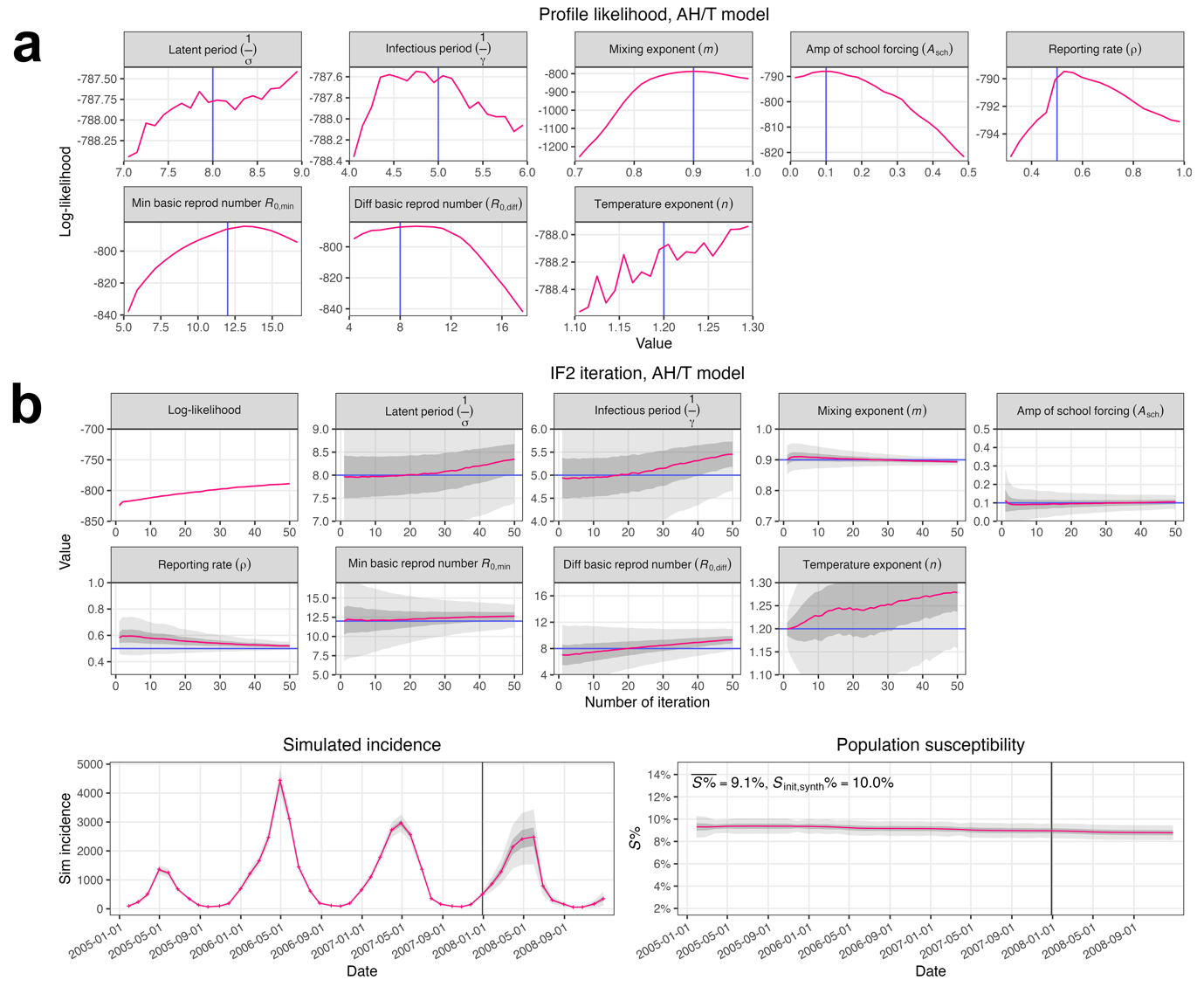


**Fig. S3** Validation of the SEIR–IF2 system using the AH/T model. (**a**) Profile likelihoods for model parameters. (**b**) IF2 iteration results.


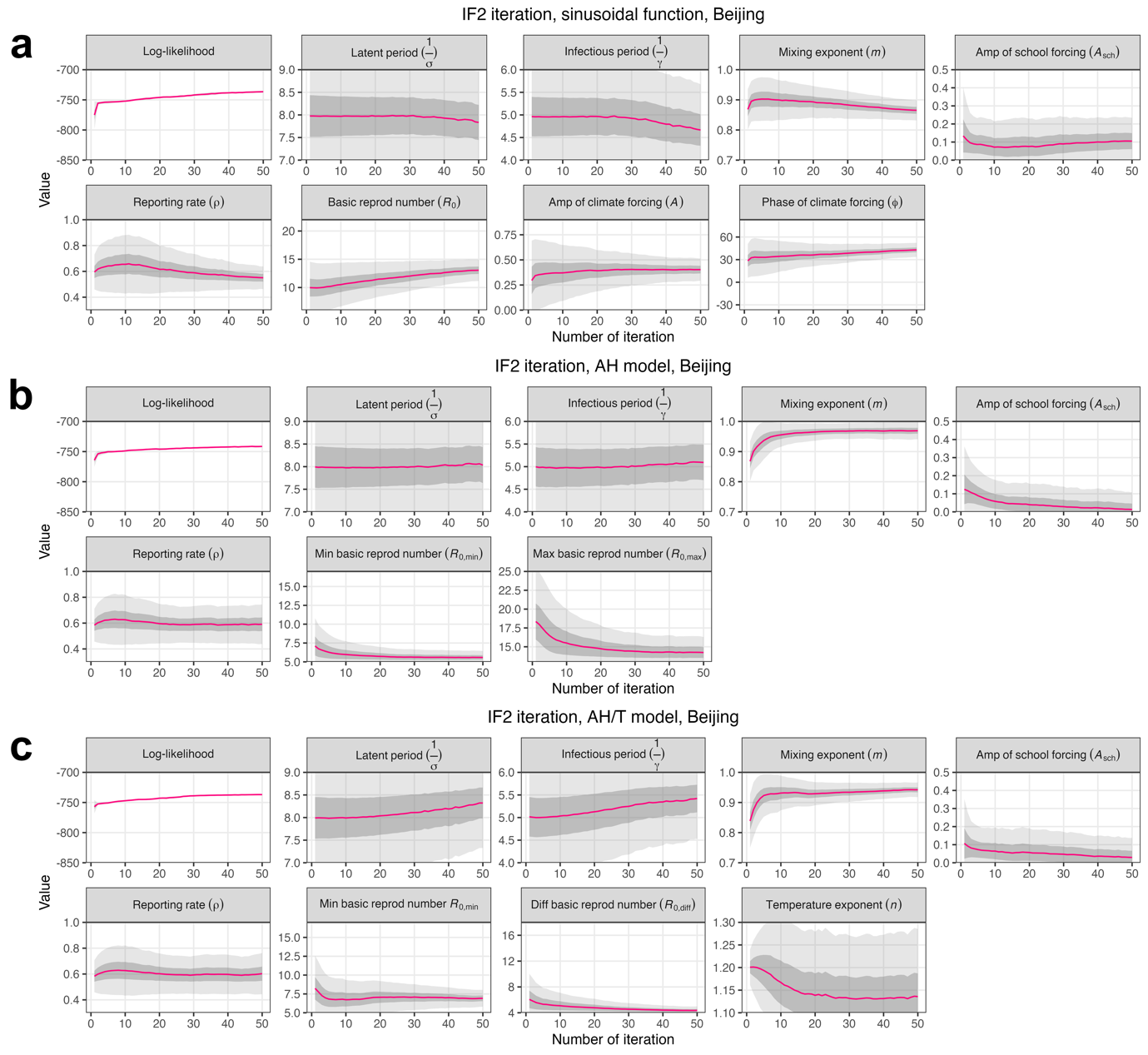


**Fig. S4** IF2 iteration results for Beijing using (**a**) the sinusoidal function, (**b**) the AH model, and (**c**) the AH/T model.


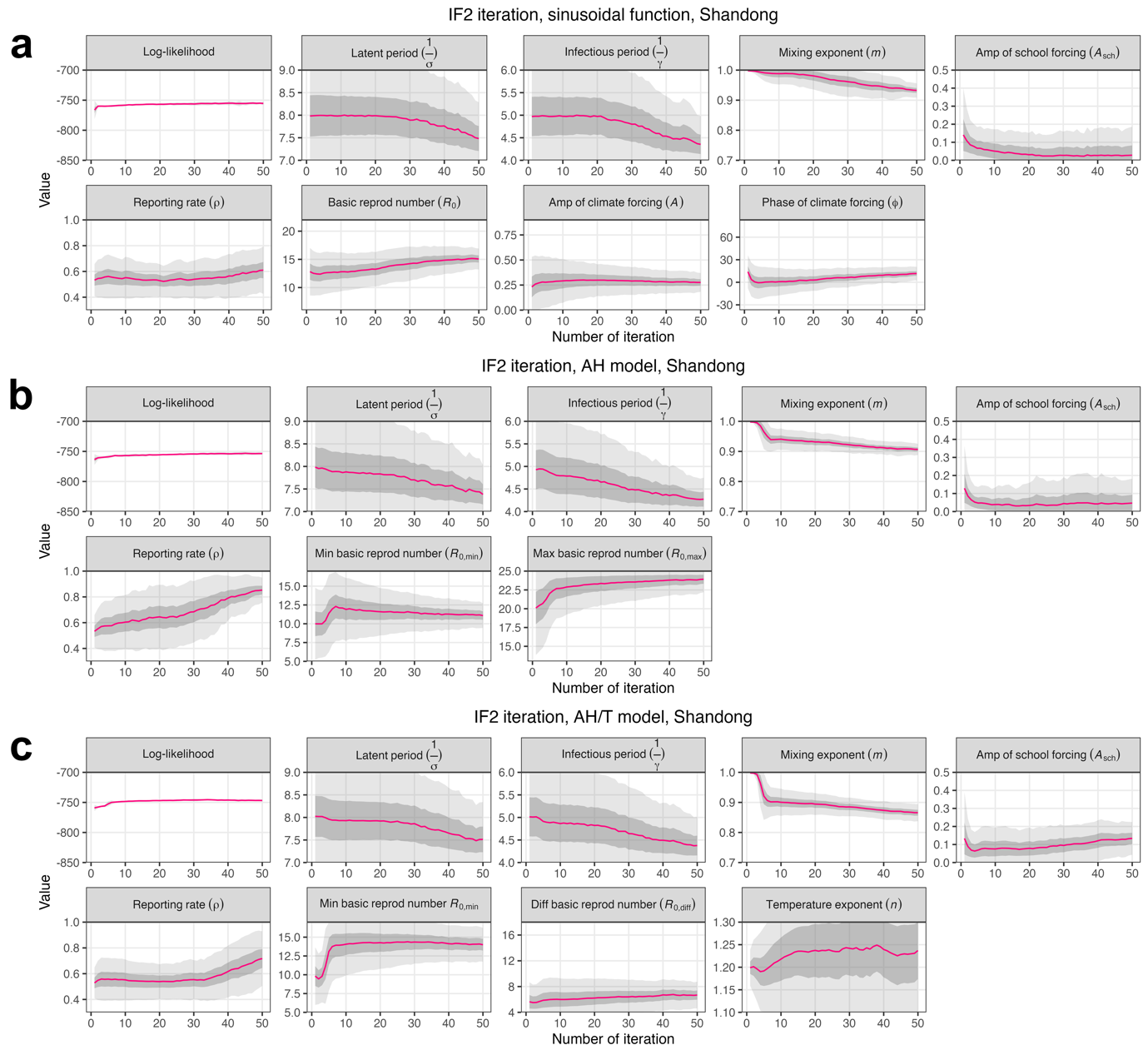


**Fig. S5** IF2 iteration results for Shandong using (**a**) the sinusoidal function, (**b**) the AH model, and (**c**) the AH/T model.


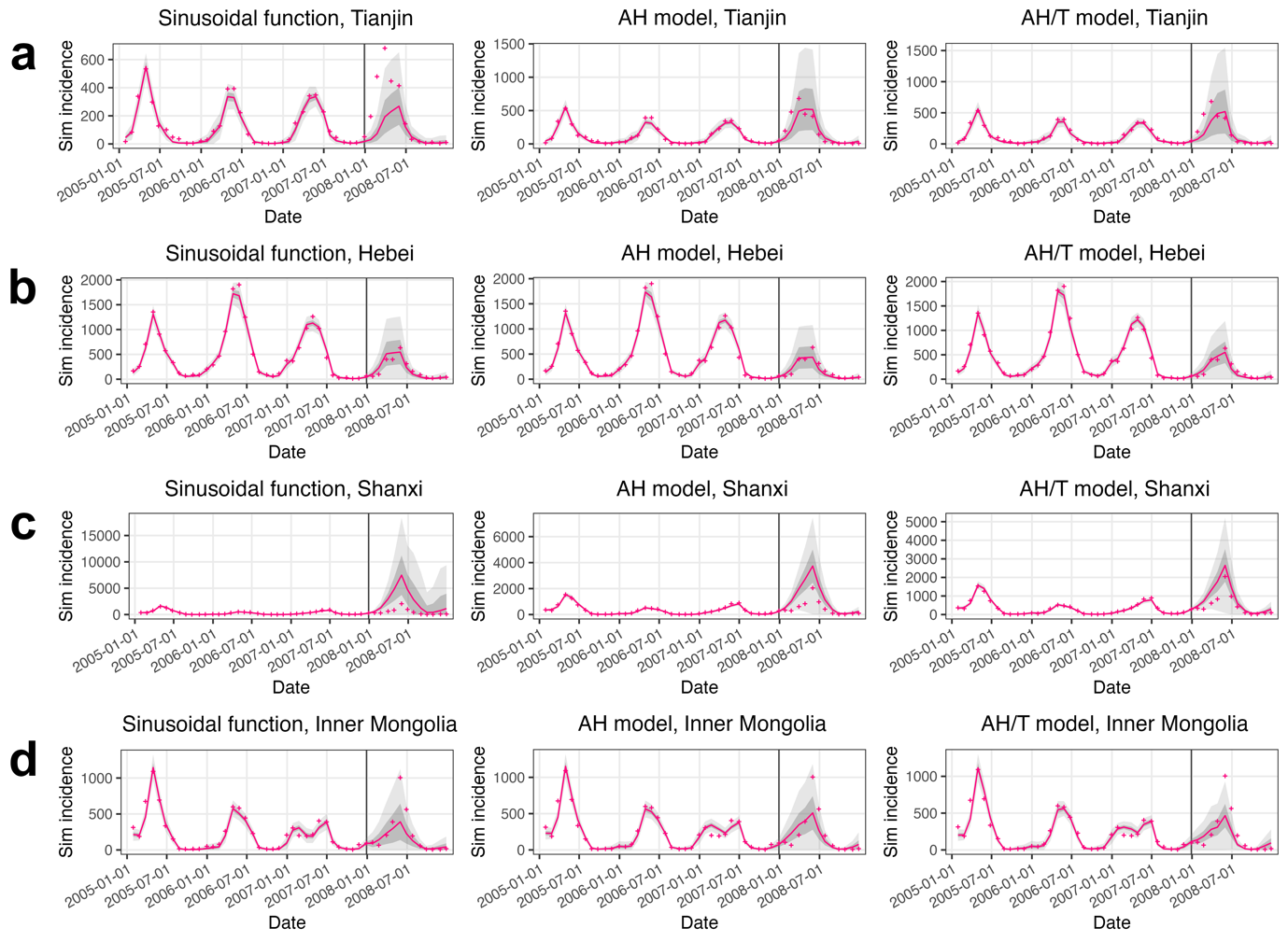


**Fig. S6** Model inference and forecasting using the sinusoidal function, the AH model, and the AH/T model, for (**a**) Tianjin, (**b**) Hebei, (**c**) Shanxi, and (**d**) Inner Mongolia in North China. Beijing, as shown in Fig. 2a–c, is also part of North China.


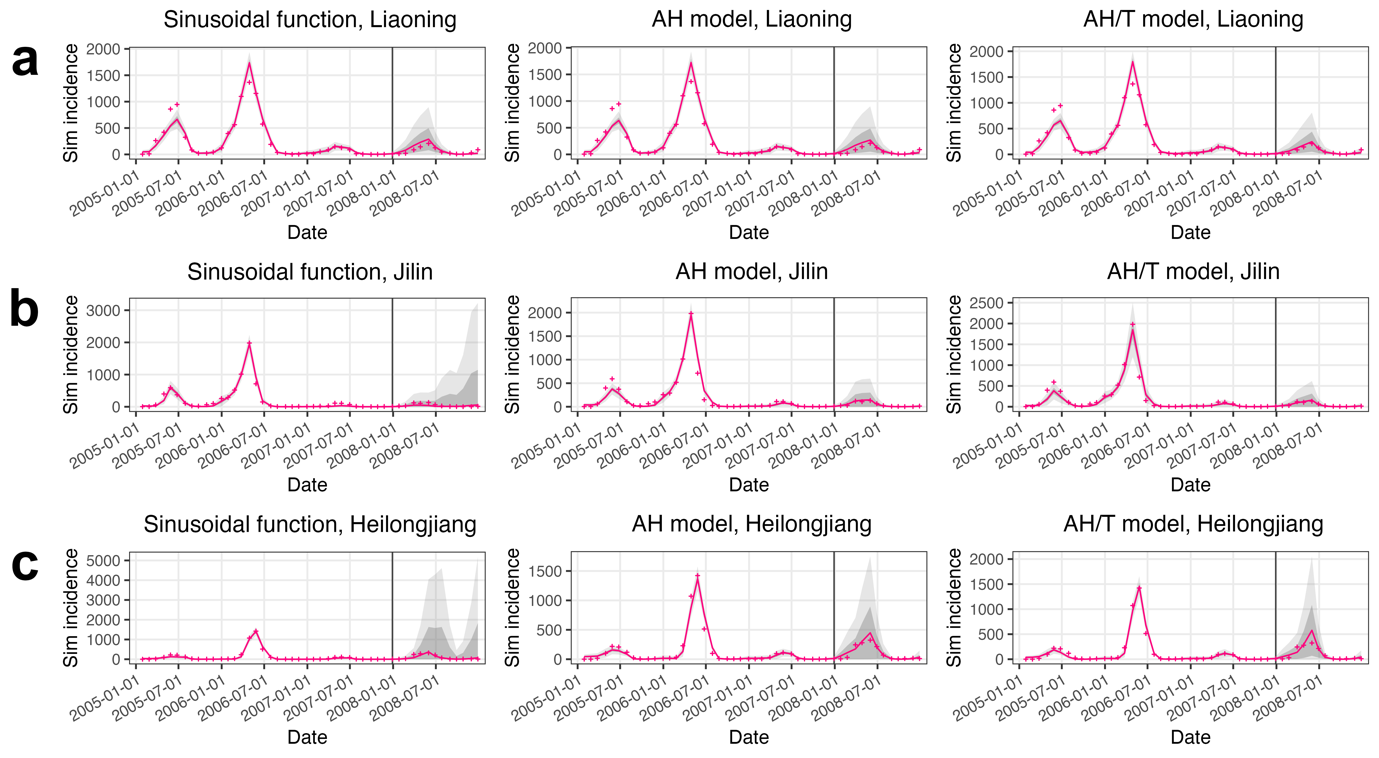


**Fig. S7** Model inference and forecasting of measles epidemic dynamics using the sinusoidal function, the AH model, and the AH/T model, for (**a**) Liaoning, (**b**) Jilin, and (**c**) Heilongjiang in Northeast China.


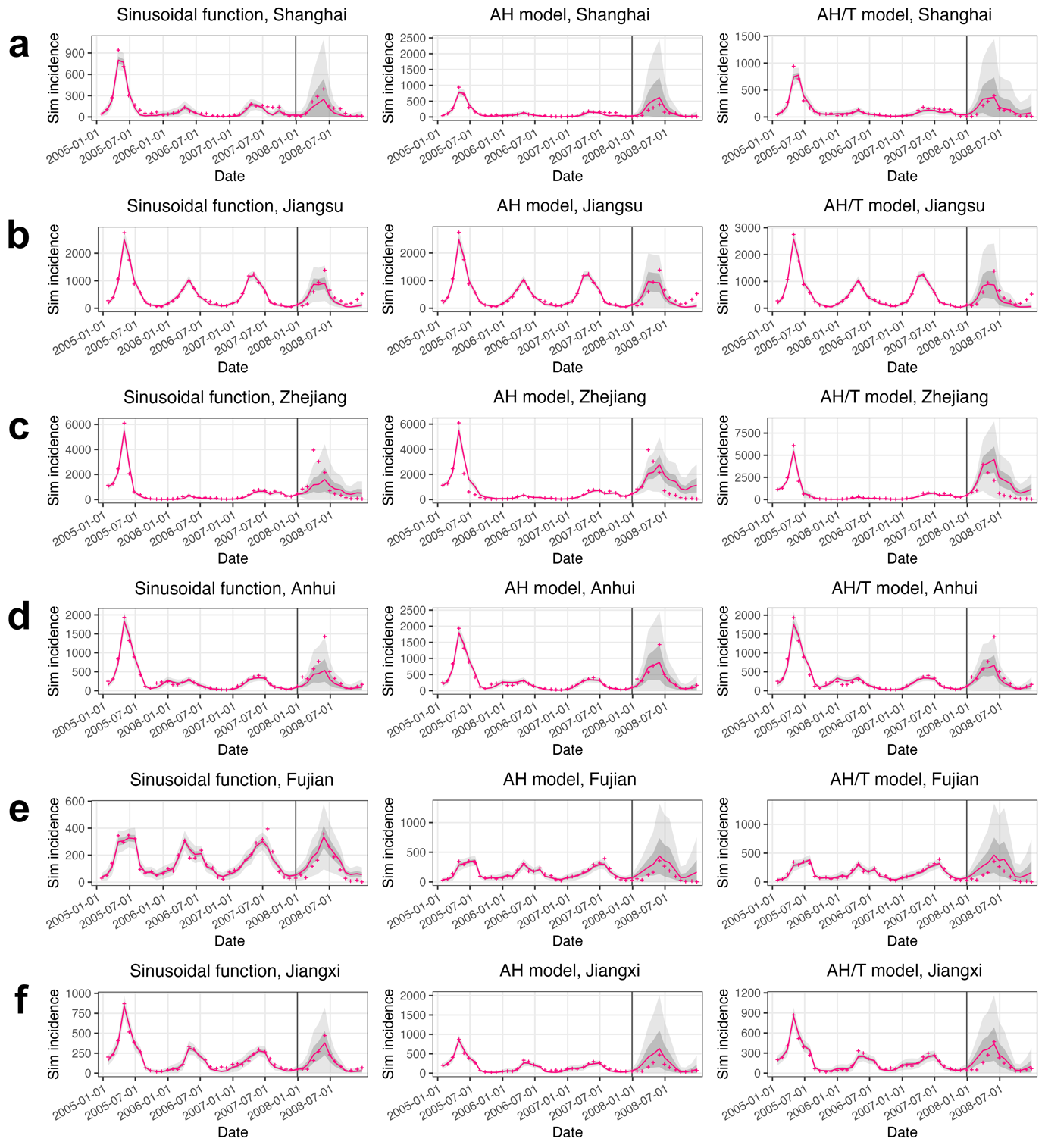


**Fig. S8** Model inference and forecasting of measles epidemic dynamics using the sinusoidal function, the AH model, and the AH/T model, for (**a**) Shanghai, (**b**) Jiangsu, (**c**) Zhejiang, (**d**) Anhui, (**e**) Fujian, and (**f**) Jiangxi in East China. Shandong, as shown in Fig. 2d–f, is also part of East China.


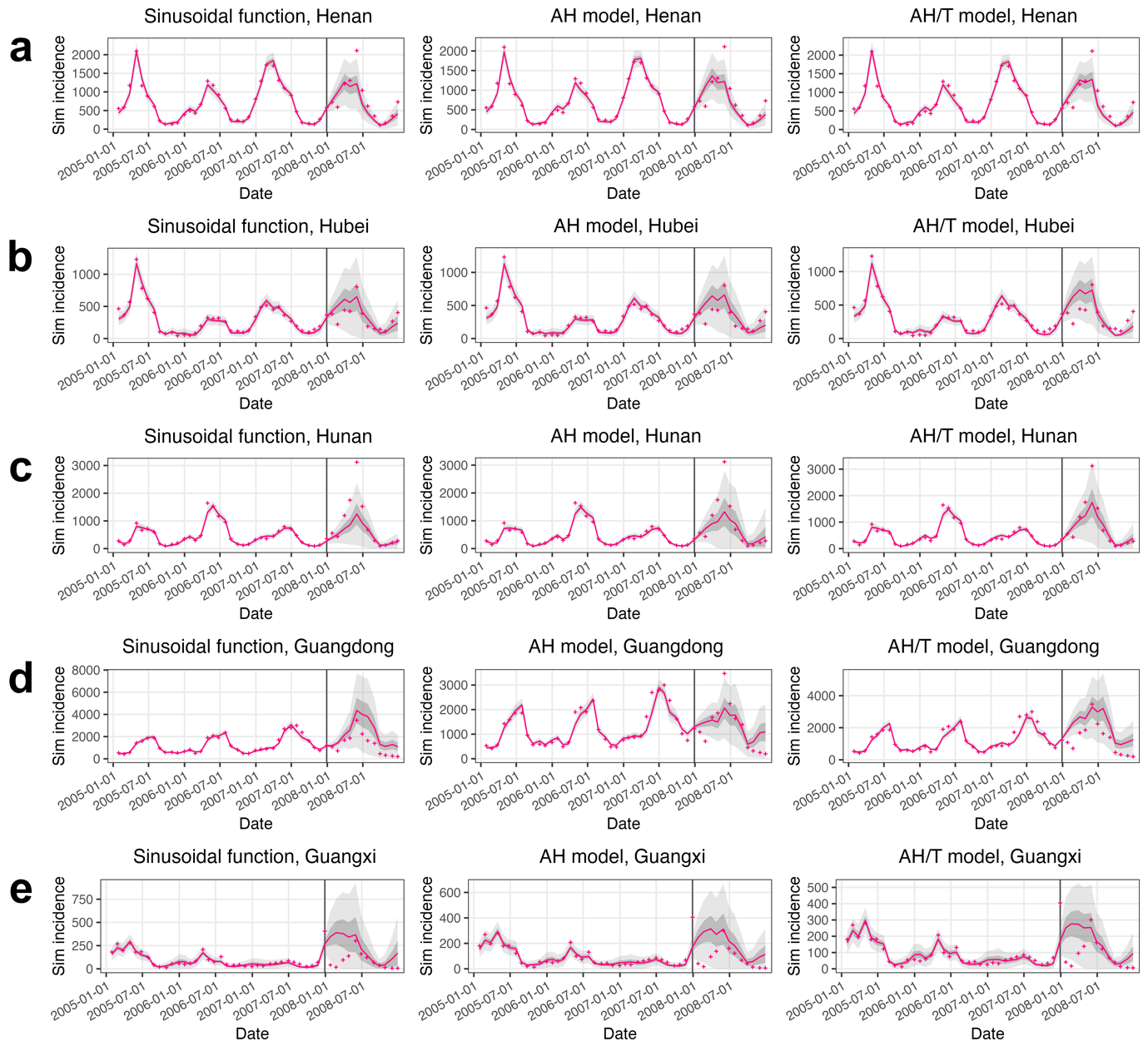


**Fig. S9** Model inference and forecasting of measles epidemic dynamics using the sinusoidal function, the AH model, and the AH/T model, for (**a**) Henan, (**b**) Hubei, (**c**) Hunan, (**d**) Guangdong, and (**e**) Guangxi in South Central China.


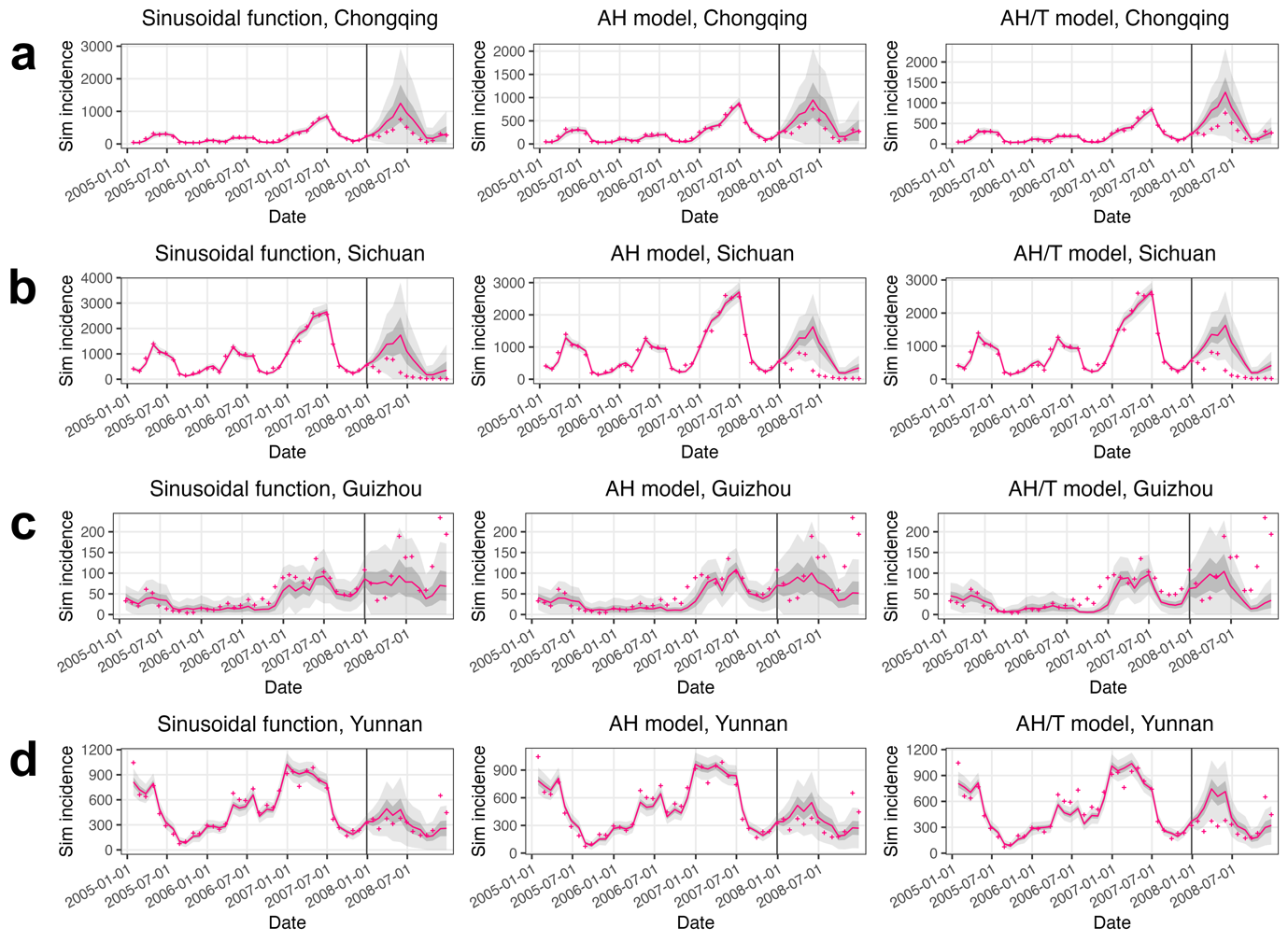


**Fig. S10** Model inference and forecasting of measles epidemic dynamics using the sinusoidal function, the AH model, and the AH/T model, for (**a**) Chongqing, (**b**) Sichuan, (**c**) Guizhou, and (**d**) Yunnan in Southwest China.


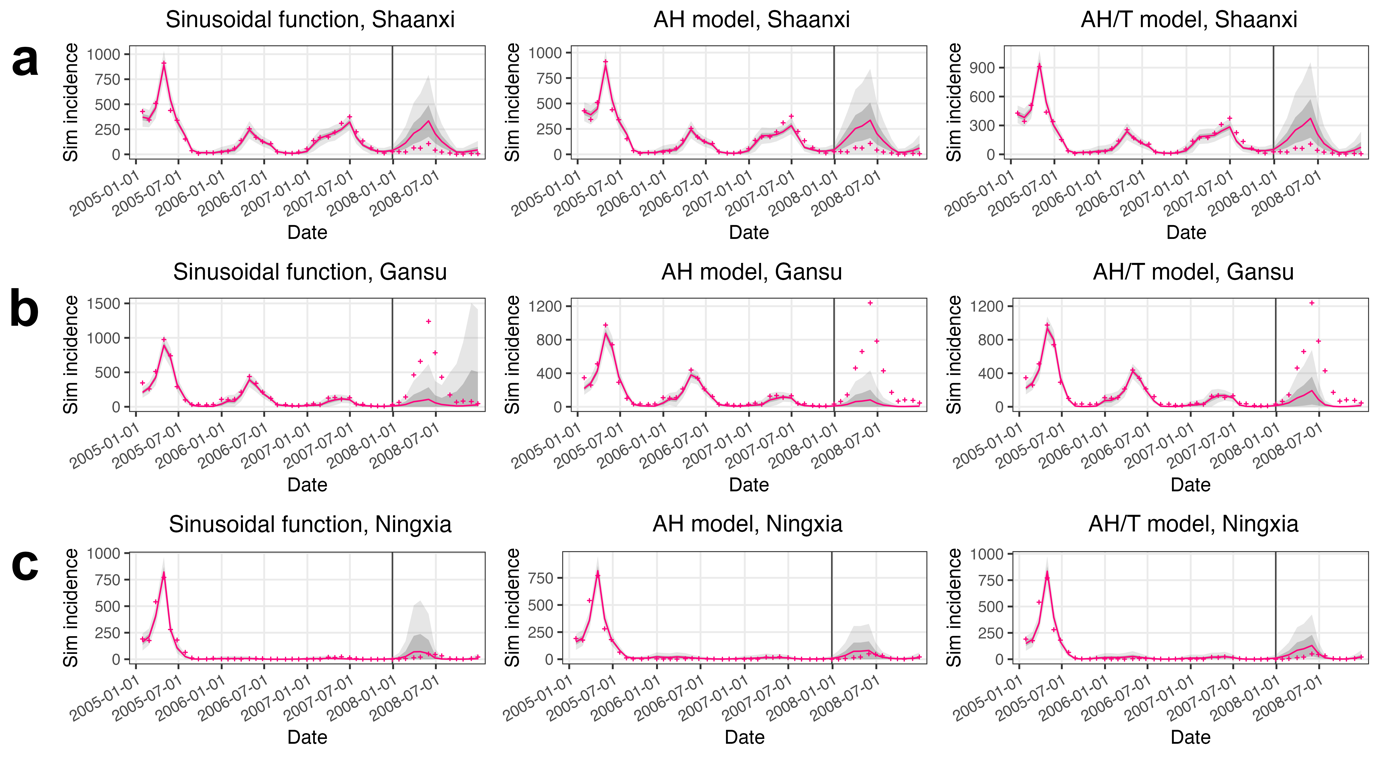


**Fig. S11** Model inference and forecasting of measles epidemic dynamics using the sinusoidal function, the AH model, and the AH/T model, for (**a**) Shaanxi, (**b**) Gansu, and (**c**) Ningxia in Northwest China.


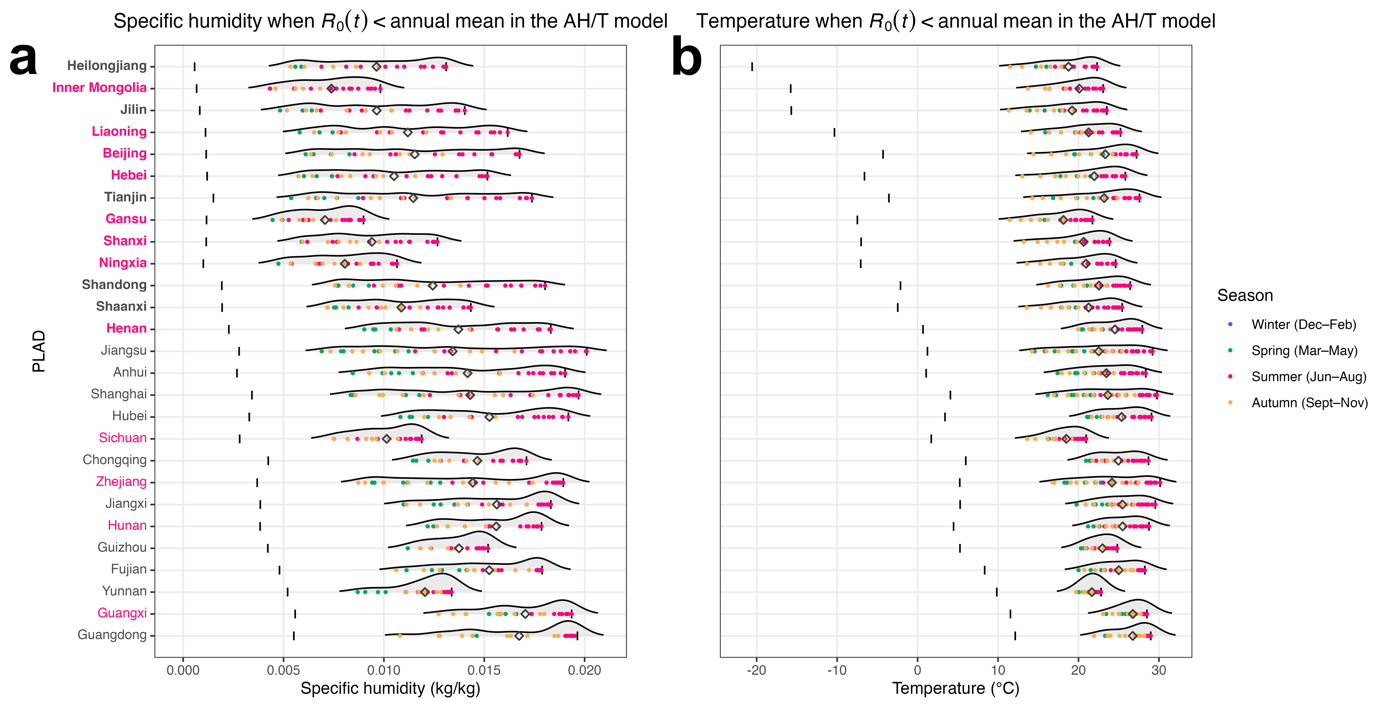


**Fig. S12** (**a**) Specific humidity levels and (**b**) temperatures when $R_{0}(t)$ were below the annual mean in the AH/T model, across PLADs in China.


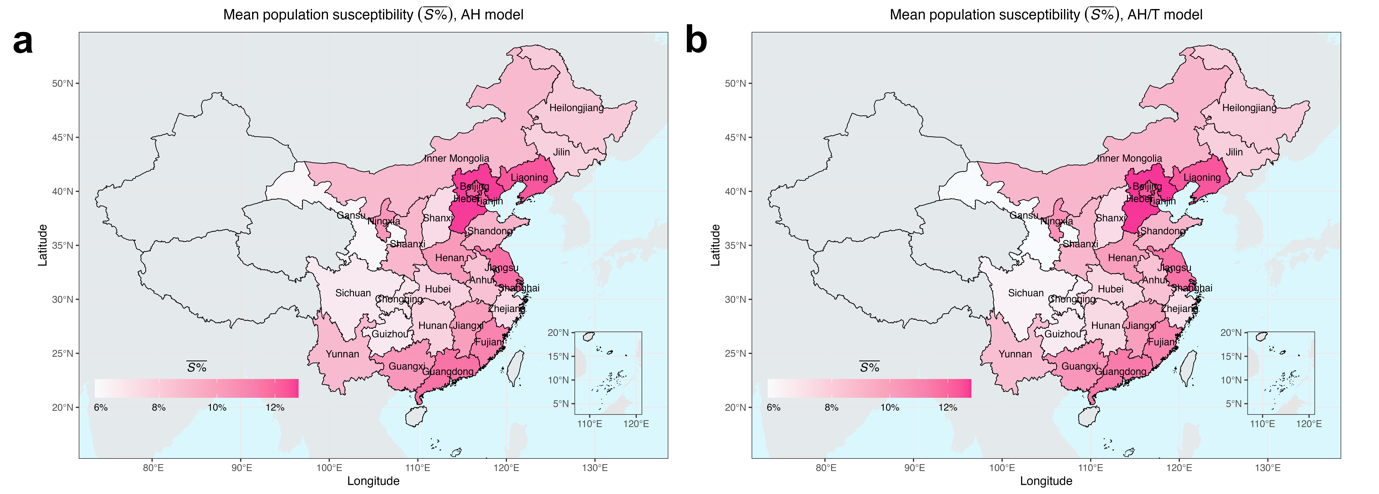


**Fig. S13** Estimated mean population susceptibility ($\bar{S\%}$) during 2005–2007 from (**a**) the AH model and (**b**) the AH/T model in PLADs of China, 2005–2007.


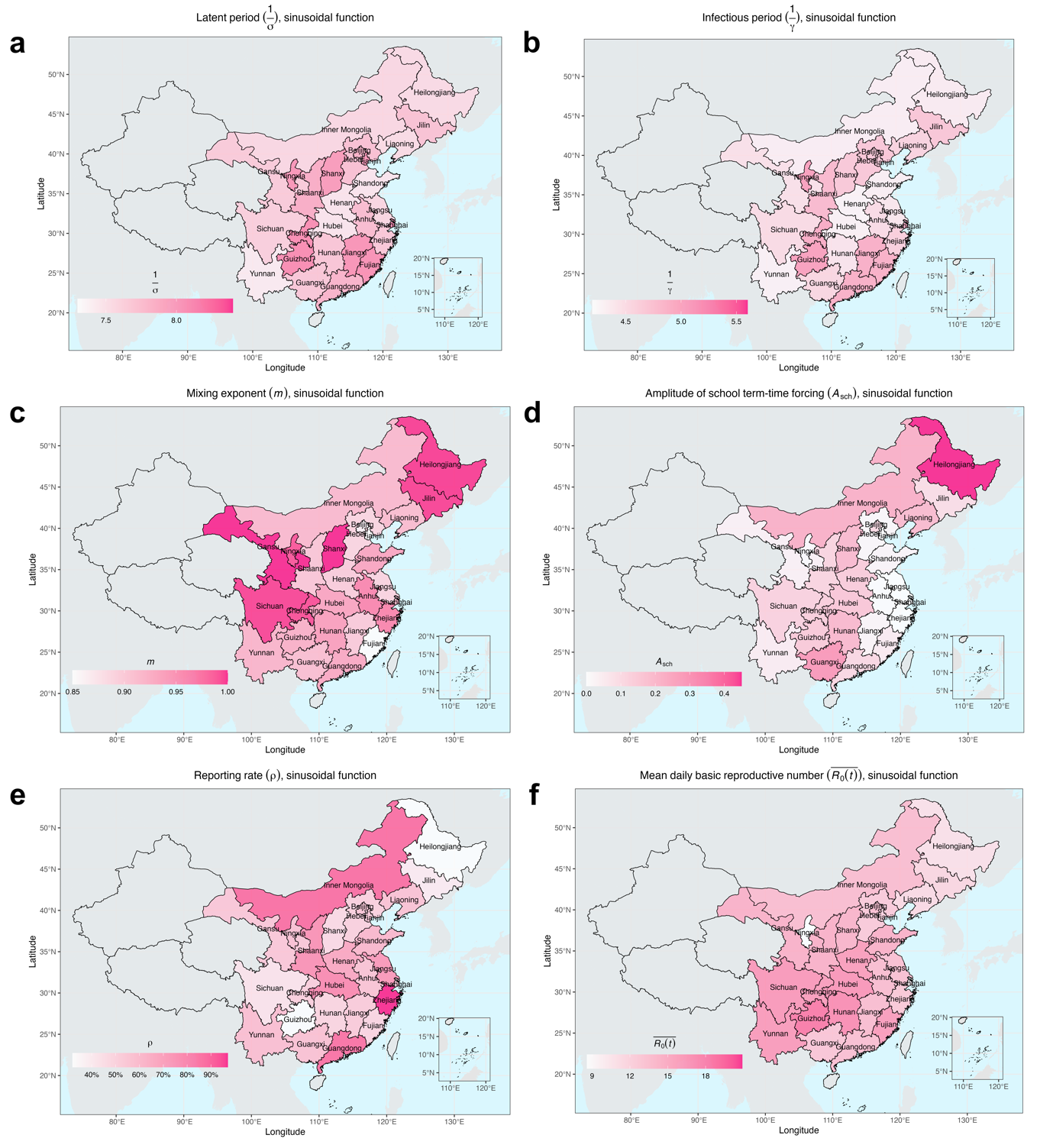


**Fig. S14** Estimated key epidemiological parameters from the sinusoidal function in PLADs of China, 2005–2007. Parameters include (**a**) latent period ($\frac{1}{\sigma}$), (**b**) infectious period ($\frac{1}{\gamma}$), (**c**) mixing exponent ($m$), (**d**) amplitude of school term-time forcing ($A_{\mathrm{sch}}$), (**e**) reporting rate ($\rho$), and (**f**) mean daily reproductive number ($\bar{R_{0}(t)}$).


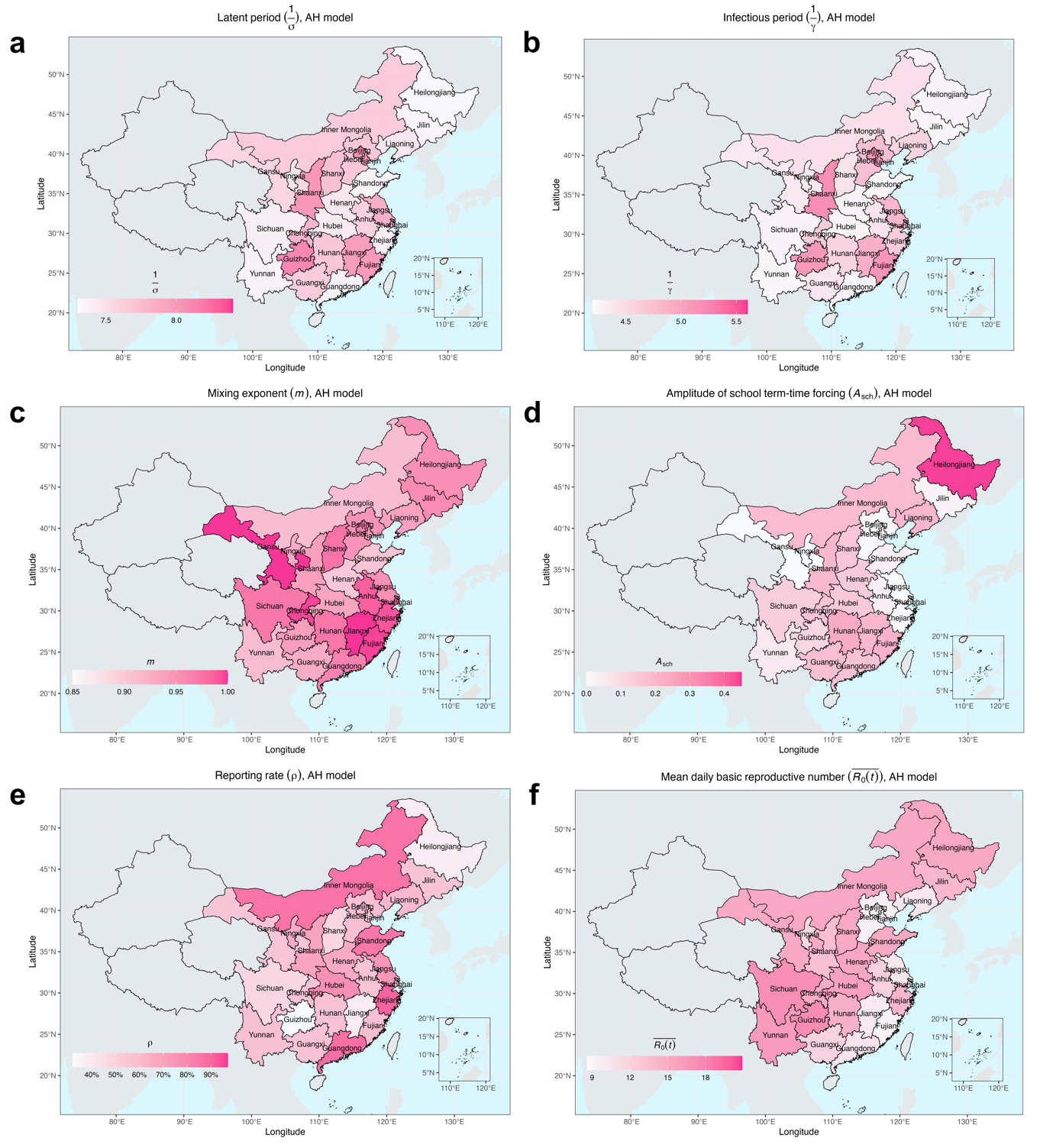


**Fig. S15** Estimated key epidemiological parameters from the AH model in PLADs of China, 2005–2007.


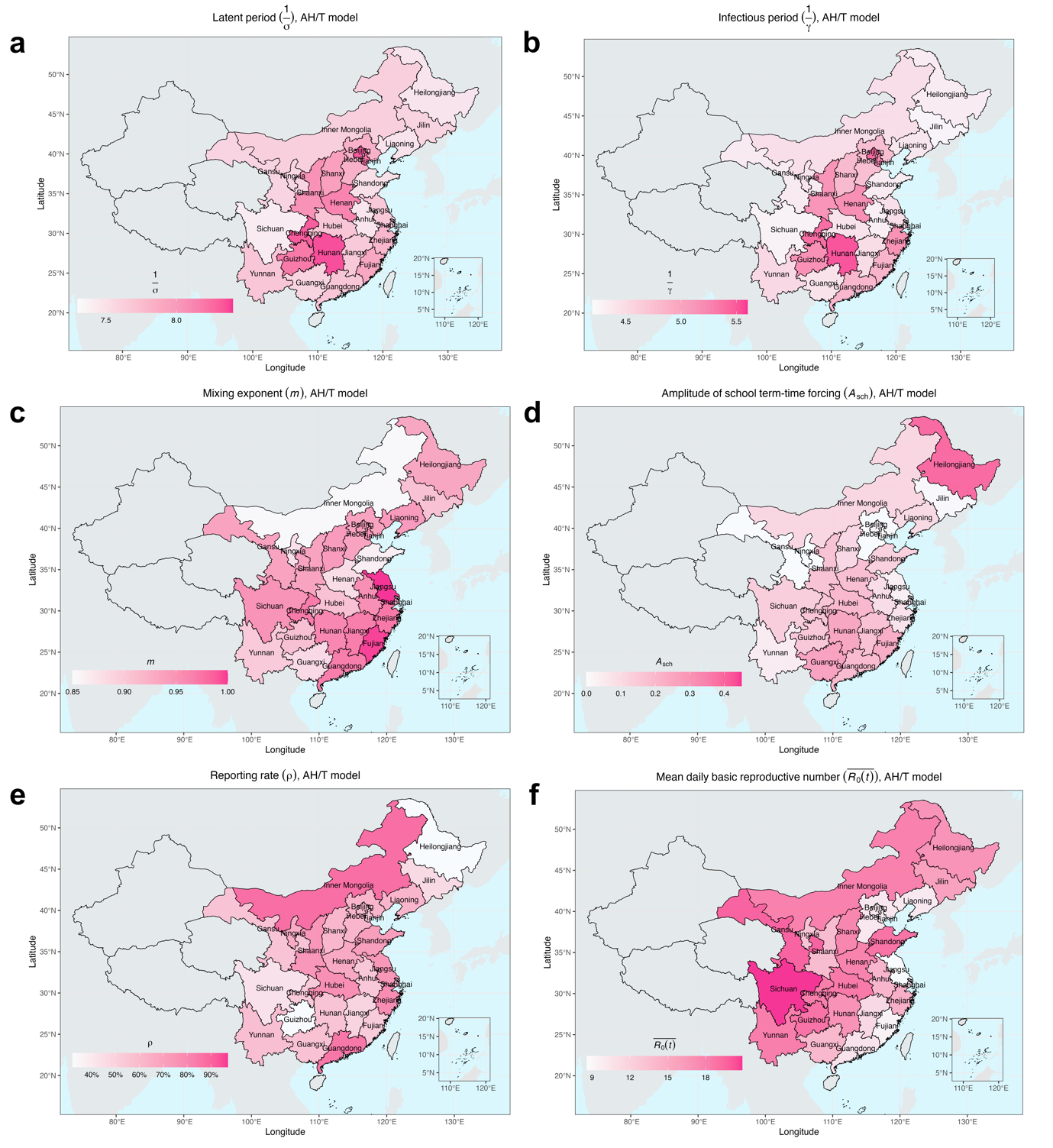


**Fig. S16** Estimated key epidemiological parameters from the AH/T model in PLADs of China, 2005–2007.


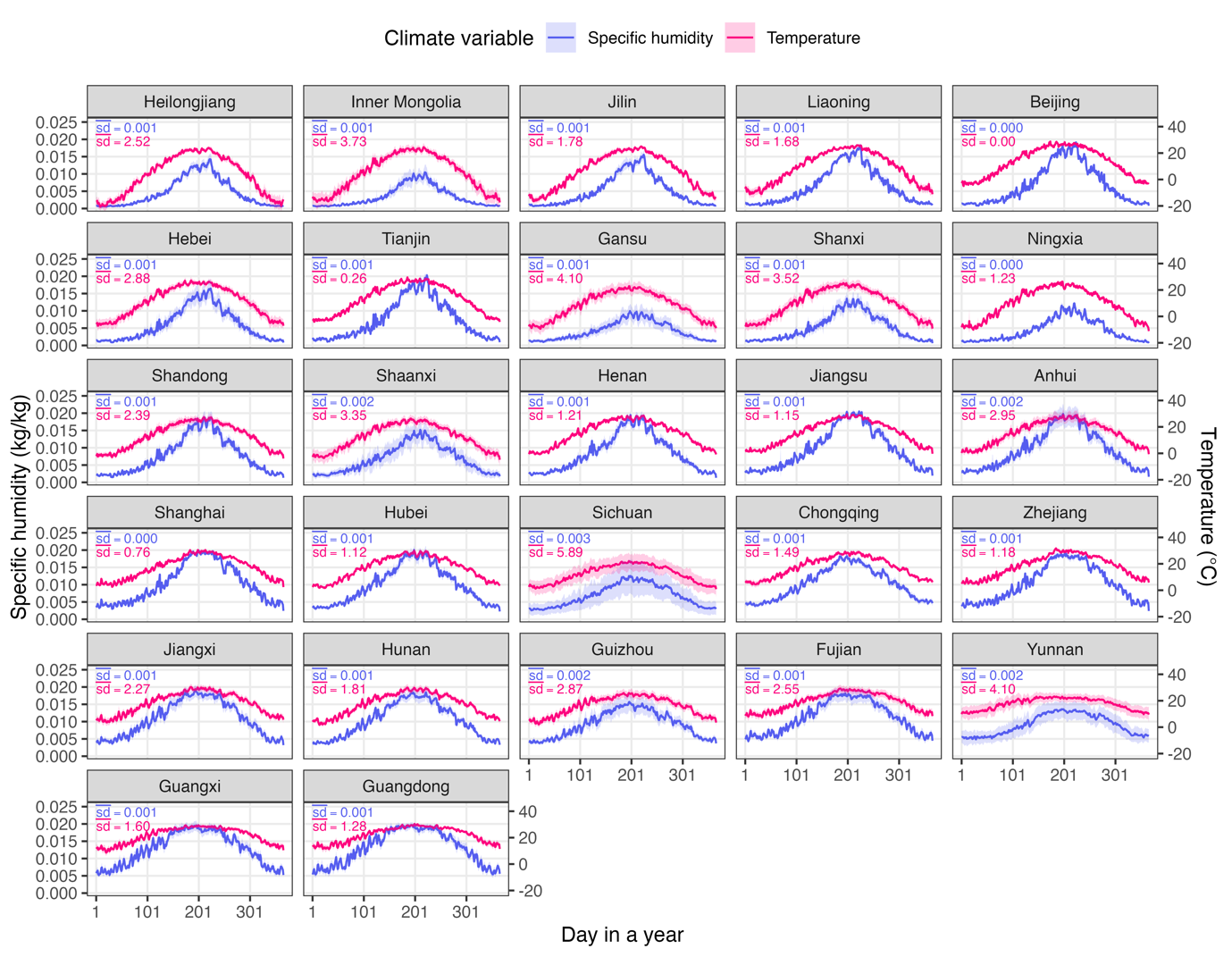


**Fig. S17** Specific humidity and temperature data collected from weather stations across cities in each PLAD during 2005–2008. Lines indicate mean values, and grey areas indicate standard deviations.


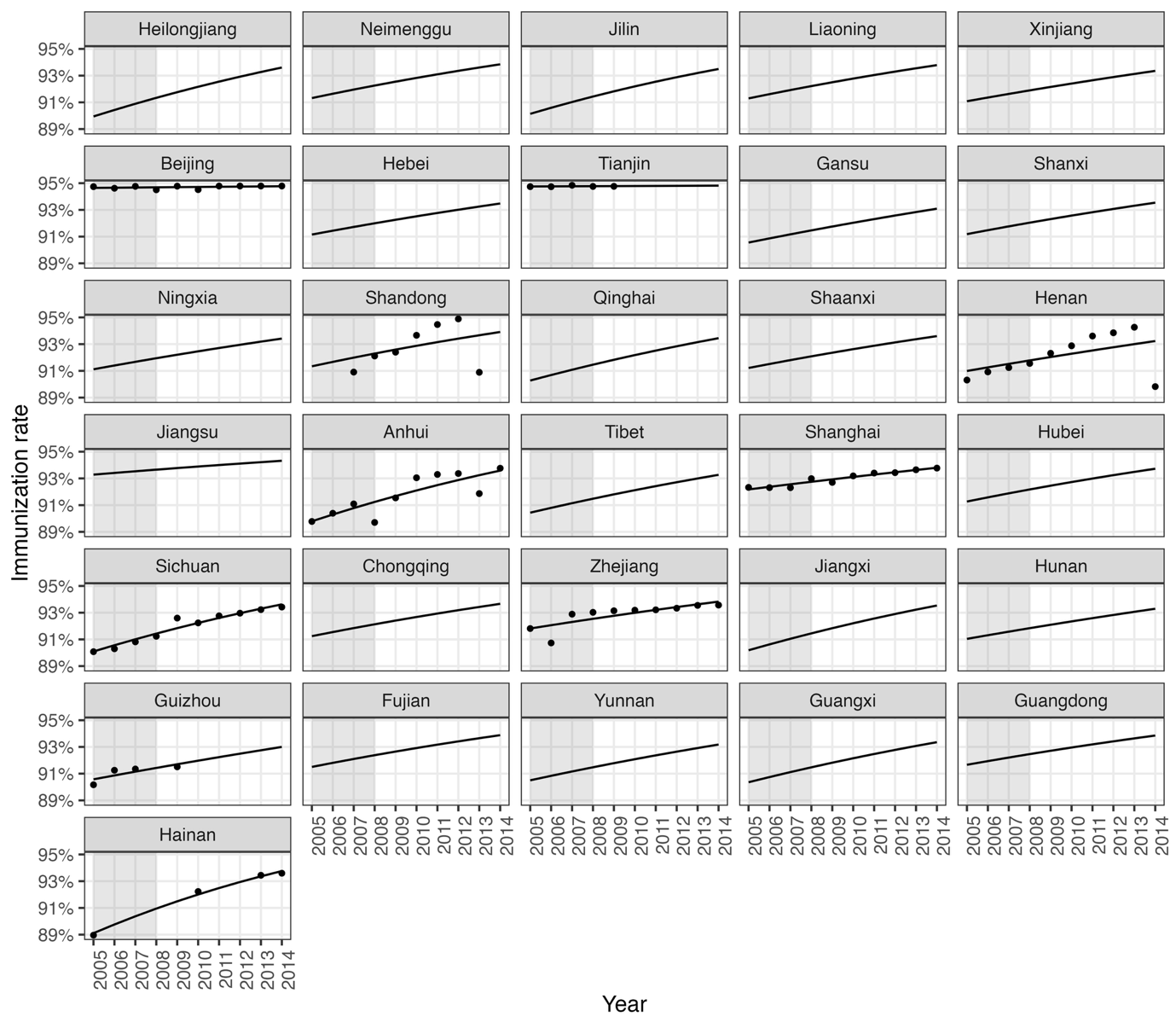


**Fig. S18** Estimated immunization rates in each PLAD during 2005–2008 (shaded areas). Dots show immunization rates computed from reported vaccination coverage data (available for 10 PLADs between 2005 and 2014). Lines show model-estimated immunization rates based on model fit to the computed rates per available vaccination data pooling all PLADs.


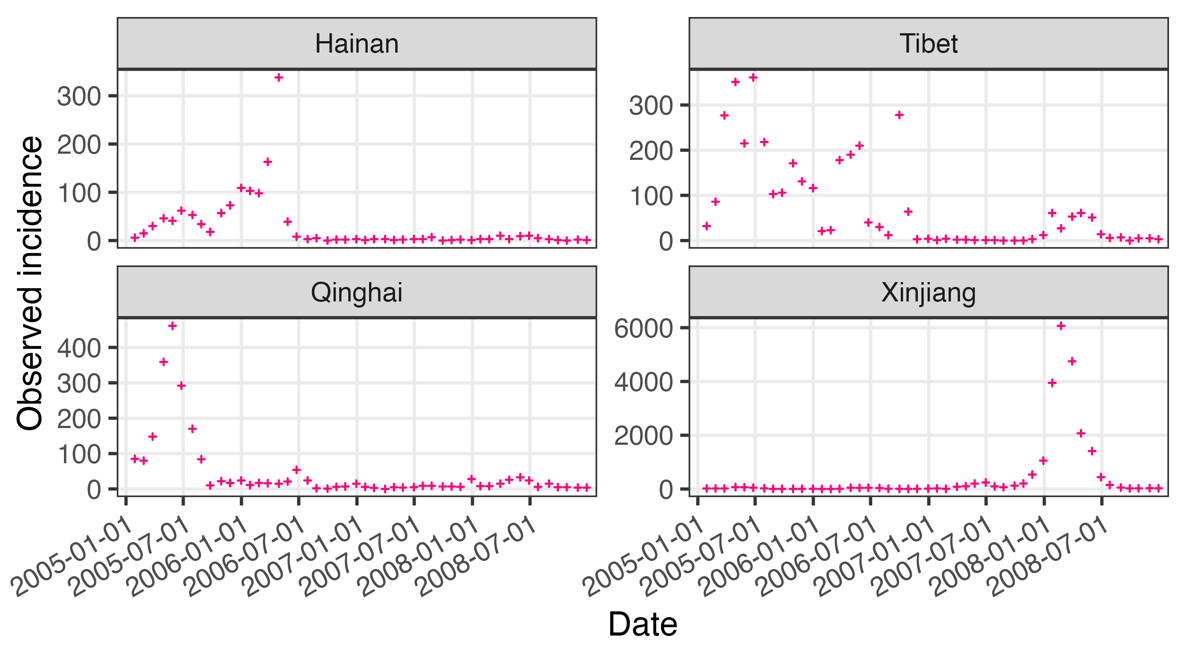


**Fig. S19** Incidence time series of four PLADs excluded from the analysis.

**Table S1** AIC values for each climate forced model and each PLAD during the inference period.

| PLAD | Sinusoidal function | AH model | AH/T model |
| --- | --- | --- | --- |
| Heilongjiang | 1470.2 | 1483.8 | 1473.3 |
| Inner Mongolia | 1526.6 | 1521.7 | 1522.3 |
| Jilin | 1513.7 | 1520.5 | 1523 |
| Liaoning | 1532.9 | 1535.9 | 1538.2 |
| Beijing | 1489 | 1496.8 | 1489.8 |
| Hebei | 1569.7 | 1570.5 | 1561.5 |
| Tianjin | 1443.9 | 1451.8 | 1453.5 |
| Gansu | 1483.1 | 1468.6 | 1461.8 |
| Shanxi | 1528.9 | 1524.8 | 1524.2 |
| Ningxia | 1438.3 | 1444.3 | 1452.1 |
| Shandong | 1526.9 | 1521.2 | 1509.6 |
| Shaanxi | 1472.8 | 1471.4 | 1472.6 |
| Henan | 1625.9 | 1626.8 | 1623 |
| Jiangsu | 1572.9 | 1572.3 | 1571.9 |
| Anhui | 1533.2 | 1530.3 | 1540.9 |
| Shanghai | 1461.9 | 1467.6 | 1475.9 |
| Hubei | 1539.5 | 1536.8 | 1530.9 |
| Sichuan | 1703.3 | 1700.9 | 1693.2 |
| Chongqing | 1494.3 | 1499.4 | 1490.2 |
| Zhejiang | 1638.1 | 1851.2 | 1634.8 |
| Jiangxi | 1459.3 | 1471.2 | 1477.4 |
| Hunan | 1569.5 | 1594.5 | 1579.8 |
| Guizhou | 1424.8 | 1419.3 | 1416.8 |
| Fujian | 1449.8 | 1475.9 | 1472.8 |
| Yunnan | 1599.8 | 1601.3 | 1607.8 |
| Guangxi | 1459.3 | 1455.9 | 1456.9 |
| Guangdong | 1753.5 | 1825.9 | 1830.7 |

**Table S2** RRMSE for each climate forced model and each PLAD during the forecast period.

| PLAD | Sinusoidal function | AH model | AH/T model |
| --- | --- | --- | --- |
| Heilongjiang | 0.624 | 0.524 | 0.791 |
| Inner Mongolia | 0.93 | 0.846 | 0.874 |
| Jilin | 1.078 | 0.524 | 1.217 |
| Liaoning | 0.822 | 0.787 | 0.52 |
| Beijing | 0.37 | 0.494 | 0.388 |
| Hebei | 0.456 | 0.449 | 0.392 |
| Tianjin | 1.011 | 0.596 | 0.724 |
| Gansu | 1.296 | 1.328 | 1.221 |
| Shanxi | 5.09 | 1.972 | 0.912 |
| Ningxia | 1.694 | 1.659 | 2.402 |
| Shandong | 0.188 | 0.504 | 0.576 |
| Shaanxi | 3.67 | 4.129 | 4.316 |
| Henan | 0.427 | 0.444 | 0.407 |
| Jiangsu | 0.532 | 0.566 | 0.562 |
| Anhui | 0.742 | 0.468 | 0.605 |
| Shanghai | 0.6 | 1.032 | 0.718 |
| Hubei | 0.463 | 0.513 | 0.62 |
| Sichuan | 2.663 | 2.598 | 2.634 |
| Chongqing | 0.956 | 0.646 | 0.973 |
| Zhejiang | 1 | 0.956 | 1.225 |
| Jiangxi | 0.327 | 1.015 | 0.646 |
| Hunan | 0.749 | 0.717 | 0.553 |
| Guizhou | 0.687 | 0.744 | 0.846 |
| Fujian | 0.469 | 1.035 | 1.267 |
| Yunnan | 0.48 | 0.509 | 0.708 |
| Guangxi | 2.255 | 1.638 | 1.49 |
| Guangdong | 0.9 | 0.514 | 0.782 |

**Table S3** Performance comparison of the sinusoidal function, the AH model, and the bimodal-AH model.

| Metric | Sinusoidal function | AH model | Bimodal-AH model |
| --- | --- | --- | --- |
| AIC_inf prd_ | 36.5% (1) | 35.1% (2) | 28.4% (3) |
| RRMSE_fcast prd_ | 44.4% (1) | 33.3% (2) | 22.2% (3) |
| *r*_fcast prd_ | 40.0% (1) | 40.0% (1) | 20.0% (3) |
| Coverage_fcast prd_ | 39.2% (1) | 37.3% (2) | 23.5% (3) |
| Peak time lag_fcast prd_ | 45.7% (1) | 34.3% (2) | 20.0% (3) |

**Table S4** Comparison of state variable and parameter estimations with previously reported ranges. Ranges within square brackets represent 95% credible intervals.

| State variable/parameter | PLAD | Sinusoidal function | AH model | AH/T model | Previously reported range |
| --- | --- | --- | --- | --- | --- |
| Mean population susceptibility, $\bar{S\%}$ | Beijing | 12.3% [11.4%, 13.2%] | 12.2% [11.3%, 13.1%] | 12.3% [11.4%, 13.2%] | 8.5% in 2007; 16.2% in 2012 (serological) (1) |
|  | Tianjin | 12.2% [11.3%, 13.1%] | 12.1% [11.2%, 13.0%] | 12.2% [11.3%, 13.1%] | 12.0% from 2011 to 2015 (serological) (2) |
|  | Jiangsu | 11.8% [10.9%, 12.8%] | 11.8% [10.9%, 12.7%] | 11.7% [10.8%, 12.6%] | 11.3% in 2008 (serological) (3) |
|  | Zhejiang | 7.9% [7.1%, 8.7%] | 7.4% [6.8%, 8.0%] | 7.3% [6.3%, 8.2%] | ~10% in 2009 (serological) (4) |
| Latent period, $\frac{1}{\sigma}$ (d) | Beijing | 7.8 [7.0, 9.0] | 8.0 [7.0, 9.0] | 8.3 [7.3, 9.0] | ~8.0 [7.0, 9.0] in 2004 (modeling) (5) |
|  | Shandong | 7.5 [7.0, 8.3] | 7.4 [7.0, 8.0] | 7.5 [7.0, 8.3] | ~8.0 [7.0, 9.0] in 2004 (modeling) (5) |
| Infectious period, $\frac{1}{\gamma}$ (d) | Beijing | 4.7 [4.0, 5.7] | 5.1 [4.0, 6.0] | 5.4 [4.5, 6] | ~5.0 [4.0, 6.0] in 2004 (modeling) (5) |
|  | Shandong | 4.4 [4.0, 5.0] | 4.3 [4.0, 4.8] | 4.4 [4.0, 5.0] | ~5.0 [4.0, 6.0] in 2004 (modeling) (5) |
| Mixing exponent, $m$ | Beijing | 0.87 [0.83, 0.90] | 0.97 [0.94, 1.00] | 0.94 [0.92, 0.97] | ~0.92 [0.90, 0.95] in 2004 (modeling) (5) |
|  | Shandong | 0.93 [0.91, 0.96] | 0.91 [0.89, 0.93] | 0.87 [0.84, 0.89] | ~0.90 [0.85, 0.95] in 2004 (modeling) (5) |
| Amplitude of school term-time forcing^a^, $A_{\mathrm{sch}}$ | Beijing | 0.11 [0.00, 0.23] | 0.01 [0.00, 0.11] | 0.03 [0.00, 0.14] | ~0.90 [0.80, 1.00] in 2004 (modeling) (5) |
|  | Shandong | 0.03 [0.00, 0.19] | 0.05 [0.00, 0.18] | 0.13 [0.04, 0.22] | ~0.60 [0.40, 0.80] in 2004 (modeling) (5) |
| Reporting rate, $\rho$ | Beijing | 55.1% [50.0%, 63.8%] | 59.1% [50.0%, 74.4%] | 60.3% [50.0%, 76.2%] | ~70% [40%, 100%] in 2004 (modeling) (5) |
|  | Shandong | 61.0% [45.0%, 79.3%] | 85.3% [75.6%, 90.0%] | 71.7% [50.5%, 90.0%] | ~50% [30%, 70%] in 2004 (modeling) (5) |

^a^ The non-age-structured SEIR model used in this study results in a lower amplitude of school term-time forcing compared to the age-structured SEIR model in the referenced modeling study.

**Table S5** Values of constant model parameters.

| Model | Parameter |
| --- | --- |
| AH/T model | Lower limit of the specific humidity ($q_{\min}$) = 3.1 g/kg (6) |
|  | Upper limit of the specific humidity ($q_{\max}$) = 18.5 g/kg (6) |
|  | Specific humidity where $R_{0,min,AH/T}$is observed ($q_{\mathrm{mi}d}$) = 10.8 g/kg (6) |
|  | Cutoff temperature ($T_{c}$) = 22.1 °C (6) |
|  | Lower limit of the temperature ($T_{\min}$) = 19.4 °C (6) |

**Table S6** Prior ranges of model state variables and parameters ($x_{0}$).

| State and parameter | | Sinusoidal function | | AH model | | AH/T model | |
| --- | --- | --- | --- | --- | --- | --- | --- |
| Bound | | Lower | Upper | Lower | Upper | Lower | Upper |
| State | Susceptible population, $S$ (7) | TSIR estimation | | TSIR estimation | | TSIR estimation | |
|  | Exposed population, $E$ | Initial incidence adjusted by reporting rate and Gaussian noise | | Initial incidence adjusted by reporting rate and Gaussian noise | | Initial incidence adjusted by reporting rate and Gaussian noise | |
|  | Infectious population, $I$ | Initial incidence adjusted by reporting rate and Gaussian noise | | Initial incidence adjusted by reporting rate and Gaussian noise | | Initial incidence adjusted by reporting rate and Gaussian noise | |
| Parameter | Latent period, $\frac{1}{\sigma}$ (d) (8) | 7 | 9 | 7 | 9 | 7 | 9 |
|  | Infectious period, $\frac{1}{\gamma}$ (d) (8) | 4 | 6 | 4 | 6 | 4 | 6 |
|  | Mixing exponent, $m$ (7) | TSIR estimation | | TSIR estimation | | TSIR estimation | |
|  | Amplitude of school term-time forcing, $A_{\mathrm{sch}}$ (5) | 0.01 | 0.5 | 0.01 | 0.5 | 0.01 | 0.5 |
|  | Reporting rate, $\rho$ (7) | TSIR estimation constrained by lower bounds ranging from 0.3 to 0.5 and upper bounds ranging from 0.6 to 1 based on the GRP rankings of PLADs | | TSIR estimation constrained by lower bounds ranging from 0.3 to 0.5 and upper bounds ranging from 0.6 to 1 based on the GRP rankings of PLADs | | TSIR estimation constrained by lower bounds ranging from 0.3 to 0.5 and upper bounds ranging from 0.6 to 1 based on the GRP rankings of PLADs | |
|  | Mean daily basic reproductive number, $R_{0}$ (9) | 8 | 20 | - | - | - | - |
|  | Amplitude of sinusoidal function, $A$ | 0.01 | 0.9 | - | - | - | - |
|  | Phase of sinusoidal function, $\varphi$ (5) | -37 (23-60) | 83 (23+60) | - | - | - | - |
|  | Minimum daily basic reproductive number prior to considering school term, $R_{0,min,AH}$ | - | - | 6 | 15 | - | - |
|  | Maximum daily basic reproductive number prior to considering school term, $R_{0,max,AH}$ | - | - | 15 | 24 | - | - |
|  | Minimum daily basic reproductive number prior to considering temperature and school term, $R_{0,min,AH/T}$ | - | - | - | - | 6 | 15 |
|  | Spread of daily basic reproductive number prior to considering temperature and school term, $R_{0,diff,AH/T}$ | - | - | - | - | 4 | 18 |
|  | Temperature exponent, $n$ (6) | - | - | - | - | 1.1 | 1.3 |

**References**

1. Zhang Z, Chen M, Wang Y, Li J, Li X, Lu L. Seroepidemiology of measles in Beijing, China: A cross-sectional study. Human Vaccines & Immunotherapeutics. 2019;15(9):2112–6.

2. Boulton ML, Wang X, Zhang Y, Montgomery JP, Wagner AL, Carlson BF, et al. A population profile of measles susceptibility in Tianjin, China. Vaccine. 2016;34(27):3037–43.

3. Liu Y, Tao H, Ma F, Lu P, Hu Y, Ding X, et al. Sero-epidemiology of measles in general population in Jiangsu province of China: Application of mixture models to interpret the results from a cross-sectional study. Vaccine. 2011;29(5):1000–4.

4. Chong KC, Rui Y, Mohammad KN, Liu Y, Zhou T, Wang MH, et al. Changes in measles seroprevalence in China after the launch of two provincial supplementary immunization activities during 2009 to 2013. The Pediatric Infectious Disease Journal. 2020;39(9).

5. Yang W, Li J, Shaman J. Characteristics of measles epidemics in China (1951–2004) and implications for elimination: A case study of three key locations. PLOS Computational Biology. 2019;15(2):e1006806.

6. Yuan H, Kramer SC, Lau EHY, Cowling BJ, Yang W. Modeling influenza seasonality in the tropics and subtropics. PLOS Computational Biology. 2021;17(6):e1009050.

7. Becker AD, Grenfell BT. tsiR: An R package for time-series Susceptible-Infected-Recovered models of epidemics. PLOS ONE. 2017;12(9):e0185528.

8. Keeling MJ, Grenfell BT. Understanding the persistence of measles: Reconciling theory, simulation and observation. Proceedings of the Royal Society of London Series B: Biological Sciences. 2002;269(1489):335–43.

9. Guerra FM, Bolotin S, Lim G, Heffernan J, Deeks SL, Li Y, et al. The basic reproduction number (R0) of measles: A systematic review. The Lancet Infectious Diseases. 2017;17(12):e420–e8.
